# Emergent collective organization of bone cells in complex curvature fields

**DOI:** 10.1038/s41467-023-36436-w

**Published:** 2023-03-03

**Authors:** Sebastien J. P. Callens, Daniel Fan, Ingmar A. J. van Hengel, Michelle Minneboo, Pedro J. Díaz-Payno, Molly M. Stevens, Lidy E. Fratila-Apachitei, Amir A. Zadpoor

**Affiliations:** 1 Department of Biomechanical Engineering, Delft University of Technology (TU Delft), Mekelweg 2, Delft, 2628CD The Netherlands; 2 Department of Materials, Department of Bioengineering, and Institute of Biomedical Engineering, Imperial College London, London, SW7 2AZ UK; 3 Department of Precision and Microsystems Engineering, Delft University of Technology (TU Delft), Mekelweg 2, Delft, 2628CD The Netherlands; 4 Department of Orthopedics and Sports Medicine, Erasmus MC University Medical Center, Rotterdam, 3015GD The Netherlands

**Keywords:** Biomedical engineering, Tissue engineering, Biophysical methods

## Abstract

Individual cells and multicellular systems respond to cell-scale curvatures in their environments, guiding migration, orientation, and tissue formation. However, it remains largely unclear how cells collectively explore and pattern complex landscapes with curvature gradients across the Euclidean and non-Euclidean spectra. Here, we show that mathematically designed substrates with controlled curvature variations induce multicellular spatiotemporal organization of preosteoblasts. We quantify curvature-induced patterning and find that cells generally prefer regions with at least one negative principal curvature. However, we also show that the developing tissue can eventually cover unfavorably curved territories, can bridge large portions of the substrates, and is often characterized by collectively aligned stress fibers. We demonstrate that this is partly regulated by cellular contractility and extracellular matrix development, underscoring the mechanical nature of curvature guidance. Our findings offer a geometric perspective on cell-environment interactions that could be harnessed in tissue engineering and regenerative medicine applications.

## Abstract (web summary)

It remains unclear how cells respond to complex extracellular geometries at the mesoscale. Here, the authors study the organization of bone cells in landscapes with varying curvatures, observing a preference for local concavities, multicellular bridging, and collective stress fiber orientation.

## Introduction

The dynamic, bidirectional interactions between cells and their intricate environment orchestrate tissue morphogenesis, homeostasis, and repair, and are implicated in numerous diseases^1–3^. The complexity of the extracellular environment is not only due to its diverse and heterogeneous composition but is also caused by its hierarchical spatial structure that imposes geometrical constraints on the force-generating cells^4,5^. Cells have long been known to sense such geometrical cues at subcellular scales^6,7^, yet recent evidence shows that geometrical features at much larger scales also affect cell migration, differentiation, and fate, as well as tissue shape and growth kinetics^8^. Unravelling this interplay between cells and the shape of their surroundings is key to advance the design of artificial scaffolds and biomaterials, where geometry can be harnessed as a micro-engineered cell cue^9–11^.

From a mathematical viewpoint, the local geometry of the extracellular environment can be fundamentally characterized using the concept of surface curvature^12^. In recent years, numerous studies have begun to address the role of cell-scale curvature on the dynamics and organization of cells and tissues. Indeed, curvature guidance has been observed in the directional migration and preferential orientation of a variety of individual cells and multicellular monolayers^13–18^. Moreover, various cell types have expressed an overall preference for local concavities as opposed to convexities^19,20^. Biophysical models have suggested key roles for cytoskeletal contractility and nuclear deformation in this large-scale curvature sensation, generally implying that cells with pronounced stress fibers avoid bending and search for relaxed configurations^21–24^. Despite the availability of such pioneering findings, it remains elusive how cells behave in more complex curvature landscapes. Early studies typically resorted to substrates with limited architectural complexity, involving cylindrical wires^13,25,26^ or isolated hemispherical substrates^27,28^, precluding many physiologically relevant geometries, including saddle shapes or sharp curvature transitions. Moreover, the mathematical descriptions of surface curvature have not received much attention, hampering the development of a unified, unambiguous theory of cell-scale curvature guidance. Indeed, many studies have considered only a single class of curved substrates^25,26^, or have relied exclusively on the concepts of convexity and concavity instead of the fundamental definitions of curvature as described by differential geometry ^20,27^.

Here, we adopt a geometry-centered perspective and demonstrate multicellular spatiotemporal organization in precise environments with varying curvature distributions. To this end, we designed several substrates, derived from mathematically defined surface families, covering a wide range of cell-scale types of curvature variation. Using high-resolution multiphoton lithography (*i.e*., a 3D printing technique with submicron resolution) and replica molding, we fabricated chips on which we cultured murine preosteoblasts for several days. Our focus on bone-like cells was motivated by the ongoing quest for geometrically optimized biomaterials that enhance bone tissue regeneration, and by the fact that these cells have been used before within the context of geometry-driven tissue growth, due to their ability to exert considerable forces on their environments (as do fibroblasts, for example) and their ability to synthesize a profound extracellular matrix at long enough time scales. While previous studies have either focused on individual cell behavior at short time scales^14,20^ or on tissue-level performance in larger-scale environments^29–31^, we studied curvature guidance at the intermediate time points where cells collectively pattern their environment and establish a template for bone-like tissue formation. By mapping 3D confocal microscopy data to the underlying curvature distributions, we explored the rules for emergent cell patterning. Specifically, we found that regions with at least one concave direction are highly attractive to cell collectives. This includes the transitions between the curved structures and their planar surroundings, which are typically ignored in other studies but explicitly considered and quantified in our work. We also found that cell collectives spontaneously detach from certain curved regions, thereby altering the extracellular geometry sensed by new cells. Moreover, we studied curvature-guided stress fiber orientation and investigated the important role of contractility in collective curvature guidance. Our results provide deeper geometric perspectives on substrate-driven multicellular organization, paving the way towards the geometric optimization of micro-engineered environments.

## Results

### Development of cell substrates with controlled curvatures

**Fig. 1:**
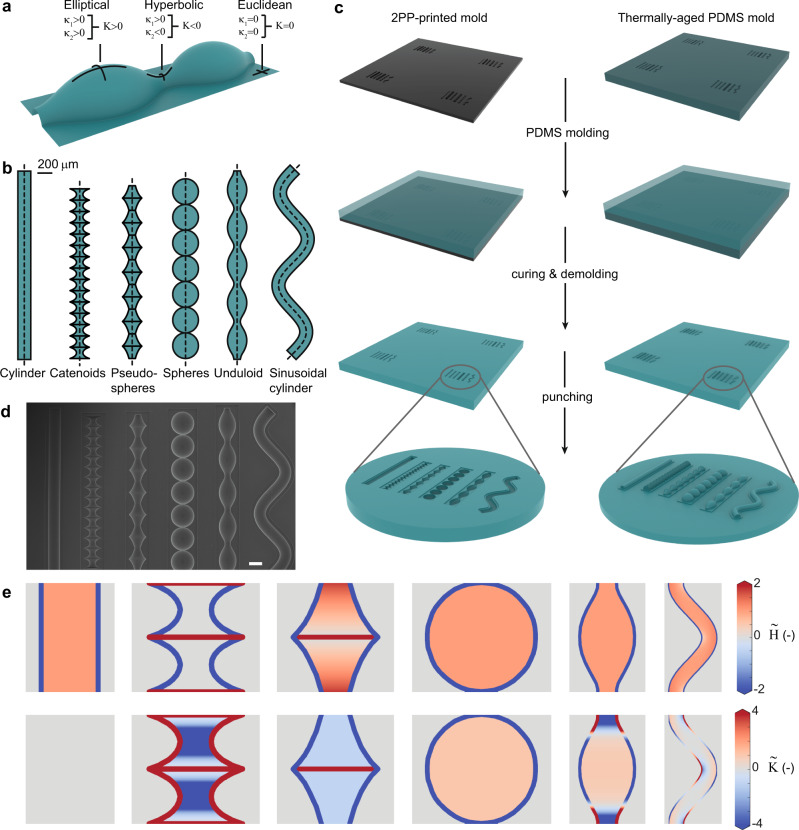
Design and microfabrication of curved cell substrates. **a** Local surface geometry defined in terms of the principal curvatures, ${\kappa }_{1}$ and ${\kappa }_{2}$, and the Gaussian curvature, $K$. **b** The surface profiles (top view) used to design curved cell substrates. The first five surfaces are surfaces of revolution (the dotted line is the rotation axis), while the last surface is obtained by sweeping a circle along a sinusoidal path (dotted line). **c** Schematic of the fabrication of the PDMS substrates with curved imprints (concave, left column) or protrusions (convex, right column). The illustration is not shown to scale. **d** SEM image of a PDMS sample with imprinted (concave) substrates. Scale bar represents 200 µm. **e** Projected curvature maps for the six types of substrates, displaying repetitive unit cells. The top and bottom rows represent the normalized mean and Gaussian curvatures, respectively. From left to right: cylinder, catenoids, pseudospheres, spheres, unduloid, and sinusoidal cylinder. The curvatures are visualized for the convex substrate variants. For the concave substrates, the mean curvatures are equal in magnitude but opposite in sign. The Gaussian curvatures remain the same.

We first set out to design substrates that would expose cells to a broad, yet controlled spectrum of curvatures. A complete description of surface curvature requires two independent curvature measures. The most common choices are either the two principal curvatures (i.e., the maximum and minimum curvatures, ${\kappa }_{1}$ and ${\kappa }_{2}$, respectively), or the pair of the mean ($H$) and Gaussian ($K$) curvatures (Fig. 1a).

1 $$H=\frac{1}{2}\left({\kappa }_{1}+{\kappa }_{2}\right)$$

2 $$K={\kappa }_{1}{\kappa }_{2}$$

We explored different surface families based on their mean and Gaussian curvature profiles, and focused on axisymmetric surfaces, as these could readily be converted to printable substrates. The first geometry that we selected was the unduloid, which is a simply periodic surface family with constant, non-zero mean curvature. An unduloid interpolates between a cylinder and a string of connected spheres, depending on its specific parametrization (Supplementary movie 1)^32^. This interpolative nature enabled us to select a cylinder, a set of spheres, and an intermediate unduloid all of which with the same (constant) mean, yet different Gaussian curvatures (Fig. 1b, e). Next, we selected two saddle surfaces (i.e., $K\, < \, 0$): the pseudosphere, having constant negative Gaussian curvature (as opposed to a sphere with constant positive Gaussian curvature), and the catenoid, having constant zero mean curvature (i.e., a minimal surface). Since these surfaces are not simply periodic by nature, we designed strings of repeating pseudospheres and catenoids, in accordance with the other surfaces (Fig. 1b). Finally, we included a sinusoidally deformed cylinder. In contrast to a normal cylinder ($K={\kappa }_{2}=0$), this deformed variant is enriched with alternating regions of positive and negative Gaussian curvatures (Fig. 1e).

We sized the surfaces to appropriate cell-scale dimensions, based on previous studies^14,20^, and used them as templates for half-revolution master molds that were 3D printed using two-photon polymerization (2PP). Single- and two-step replica molding with poly(dimethylsiloxane) (PDMS) provided us with precisely curved cell culture environments, consisting of both concave imprints ($H \, < \, 0$) and convex protrusions ($H \, > \, 0$) of the same surfaces (Fig. 1c, d and Supplementary Figs. 1 and 2). By using both the concave and convex variants, we could significantly expand our total curvature spectrum, as these substrates feature the same Gaussian curvatures, yet opposite mean curvatures. A natural consequence of using substrates consisting of half-revolution surfaces attached to planar surroundings is that the curved-to-planar transitions present relatively high local curvature changes (Fig. 1e). While typically ignored in previous studies, we explicitly account for these regions in our subsequent analysis, which is enabled by the analytical parametrizations of the surface families used in this study (Supplementary note 1).

### Murine preosteoblasts prefer regions with negative minimum principal curvature

**Fig. 2:**
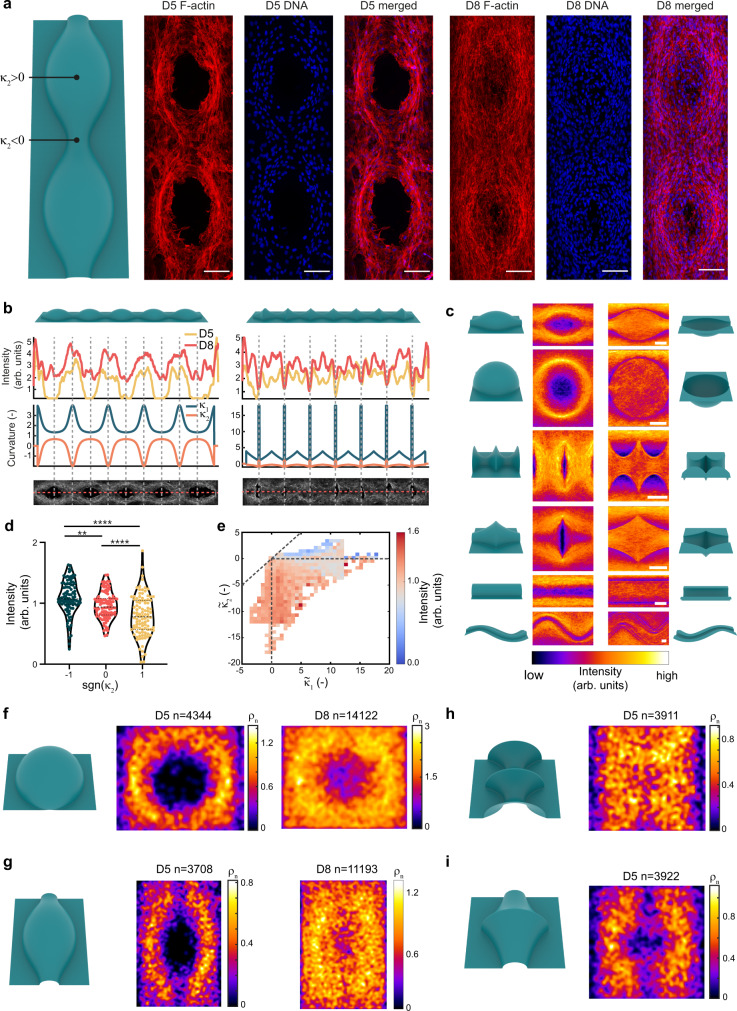
Multicellular spatiotemporal patterning on curved substrates. **a** Representative maximum intensity projections of the F-actin and DNA immunostainings for the convex unduloid at day 5 (D5) and day 8 (D8). Scale bars are 100 µm. **b** Normalized actin intensity, measured along the centerline, varies as a function of the principal curvatures ${\kappa }_{1}$ and ${\kappa }_{2}$. The intensity profiles are obtained from multiple specimens (*n* ≥ 3) for the convex unduloid (left) and convex pseudosphere (right) substrates (see also Supplementary Fig. 3a). **c** Frequency maps displaying spatial actin patterning on day 8. The data is obtained by stacking periodic units from multiple images (*n* > 15 from at least three independent experiments) for both the convex (left column) and concave (right column) variants (see also Supplementary Fig. 3b–d). Scale bars are 100 μm. **d** Normalized actin intensity versus the sign of ${\kappa }_{2}$ for convex substrates on day 8. 100 random data points for each category were sampled from all the available data (superpixels of 80×80 pixels). The data are shown as violin plots with median and interquartile range. Brown-Forsythe and Welch’s one-way ANOVA with Games-Howell’s multiple comparisons: **p = 0.0020, ****p < 0.0001. **e** Heat map of the median normalized actin intensity vs. the two normalized principal substrate curvatures ${\kappa }_{1}$ and ${\kappa }_{2}$ for all substrates on day 8. The data points are obtained by rasterizing the projected confocal images in elements of 20 by 20 pixels. **f**–**i** Frequency maps of the nuclei centroids (${\rho }_{n}$) at day 5 or day 8, obtained using data from at least three independent experiments (n in the figures indicates number of nuclei). The maps were obtained by convolving a 2D histogram of all centroid positions (100 × 100 bins) with a Gaussian filter (3 × 3, σ = 1.5). Source data are provided as a Source Data file.

To investigate curvature-guided spatiotemporal cell patterning, we cultured murine preosteoblasts (MC3T3-E1) on the curved substrates for several days. This cell line has been used before in the context of curvature-driven tissue growth^31,33^. After 5 days, we observed confluent layers on the planar regions and curvature-dependent patterning on the non-planar regions. After 8 days, this trend continued and large cell collectives were found to differentially cover the substrates (Fig. 2a and Supplementary Fig. 3a). By inspecting cell coverage using F-actin frequency maps, created by superimposing confocal image projections, we found strong differences in the patterning on the concave ($H \, < \,0$) and convex ($H \, > \,0$) variants of the substrates (Fig. 2c and Supplementary Fig. 3b–d). Uniform coverage was observed in the concave substrates, while the convex variants exhibited distinct regions with high and low actin intensities. On these convex substrates, we found more coverage on the hyperbolic regions (saddle-shaped, $K \, < \,0$) than on the elliptical regions (sphere-like, $K \, > \,0$), as exemplified on the unduloid substrate with a constant mean and varying Gaussian curvatures (Fig. 2a–c). On the convex catenoids and pseudospheres ($K \, < \,0$), we observed full cell coverage along the entire substrate, except for the sharp (locally elliptic) transition regions between the saddles. Moreover, we consistently found relatively high intensities at the transition regions between the convex structures and their planar surroundings, while the reverse was observed at the concave-to-planar transitions. These observations point towards the collective preference of the cells to pattern regions where the minimum principal curvature is negative (i.e., ${\kappa }_{2} \, < \,0$), which includes all regions with at least one concave direction (Fig. 2d). This translates to regions with either $K \, < \,0$ (saddle shapes), or $K\ge 0$ combined with $H \, < \,0$ (e.g., concave spheres). Indeed, the convex-to-planar transitions, that feature strong cell attraction, are also characterized by ${\kappa }_{2} \, < \,0$. When considering the mean actin intensity across the full curvature spectrum presented to the cells, higher mean intensities were observed in regions with a negative minimum principal curvature (Fig. 2e). Cell coverage on the curved substrates could also be assessed by considering the spatial distribution of the cell nuclei. Frequency maps of the nuclei centroids on the convex spherical and unduloid substrates, capturing the distribution of thousands of nuclei, revealed that the nuclei are preferentially situated close to the regions with ${\kappa }_{2} \, < \,0$ (Fig. 2f, g). This effect is more apparent on day 5, and diminishes on day 8, in line with the observations regarding the F-actin distribution. As expected, the cell nuclei cover the saddle-shaped substrates (Fig. 2h, i) more uniformly than the substrates with ${\kappa }_{2} \, > \,0$, yet nuclei avoid the locally elliptic transitions between the saddles (especially for the pseudospheres in Fig. 2i). These nuclei distribution maps, therefore, further confirm the general preference of the cells for regions with negative values of the minimum principal curvature. Nevertheless, the frequency maps and the intensity plots also show that cells do not entirely avoid unfavorably curved regions. For example, substantial regions of the spherical substrates were covered with cells at day 8, despite the constant positive ${\kappa }_{2}$, suggesting a collective ability to conquer such less favorable curvatures at longer time points.

### Distance to ${{{{{{\boldsymbol{\kappa }}}}}}}_{{{{{{\boldsymbol{2}}}}}}} \, < \, {{{{{\boldsymbol{0}}}}}}$ and curvature magnitude characterize spatial cell patterning

**Fig. 3:**
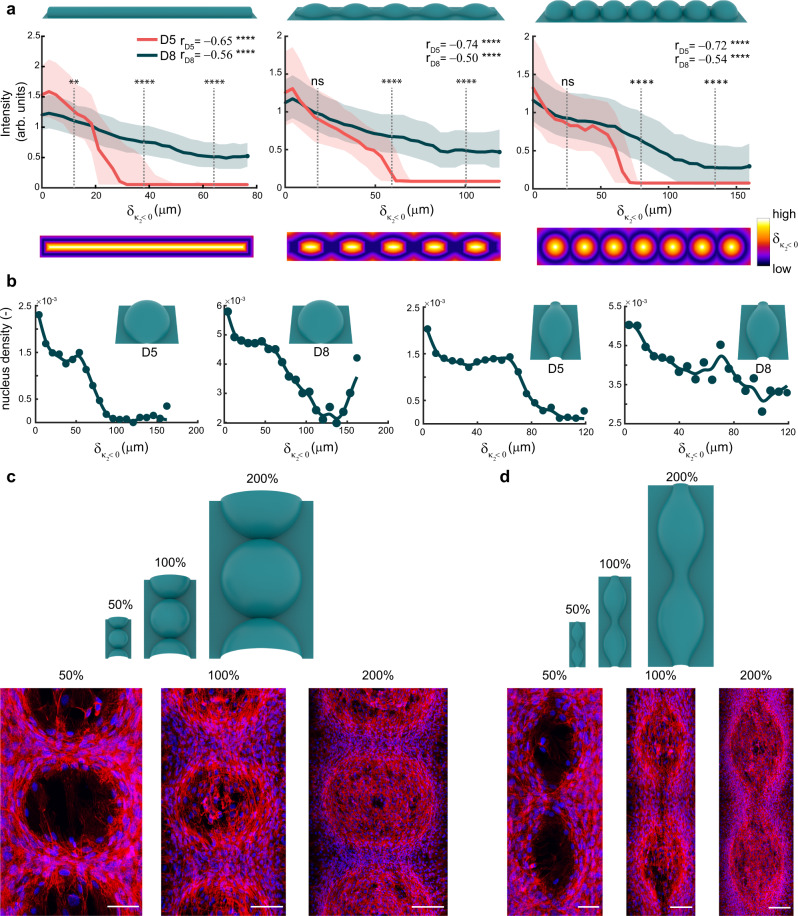
Patterning as a function of ${\delta }_{{k}_{2} < 0}$ and substrate scale. **a** Normalized F-actin intensity versus ${\delta }_{{\kappa }_{2} < 0}$ for three convex substrates at day 5 and 8. The solid line represents the median value, while the shaded areas correspond to the interquartile range. The r-values represent the Spearman’s correlation coefficients. Two-tailed Mann–Whitney *U* tests: **p = 0.0025, ****p < 0.0001, ns = not significant. The bottom row depicts the Euclidean distance maps for the three considered (convex) substrates. Combined data from *n* = 4 independent experiments for D8, *n* = 3 for D5 spheres and unduloid, *n* = 1 for D5 cylinder (representative of 3 cylinders). **b** Nucleus density (number of nuclei at a given ${\delta }_{{\kappa }_{2} < 0}$ normalized by number of regions with that value of ${\delta }_{{\kappa }_{2} < 0}$) versus ${\delta }_{{\kappa }_{2} < 0}$ for the day 5 and day 8 data presented in Fig. 2f, g. The curve through the data points is a smoothing spline. **c** Representative maximum intensity projections of merged F-actin (red) and DNA (blue) immunostainings at day 8 on scaled versions of the convex spherical substrates (100% being the original scale). **d** The same as in **c** but for the scaled convex unduloid substrate. Scale bars are 50 µm (50% scale), 100 µm (100% scale), and 200 µm (200% scale). Source data are provided as a Source Data file.

We hypothesized that the presence of cells on the convex regions with ${\kappa }_{2}\ge 0$, which was more apparent on day 8 than on day 5, was caused by a collective crowding effect, whereby cells expand from preferentially curved regions into less favorable territory. To investigate this, we created distance maps, quantifying the shortest distance to a region with ${\kappa }_{2} \, < \,0$ for every point on the substrate. These distance maps are relevant for the substrates that contain substantial regions with ${\kappa }_{2}\ge 0$, such as the convex cylinder, unduloid and spherical substrates. On those substrates, we observed that the distance maps closely resemble the spatial distribution of cells. This observation was quantified by plotting the normalized intensity versus the distance value (termed ${\delta }_{{\kappa }_{2} < 0}$), demonstrating a reduction in intensity for increasing distance (Fig. 3a). The effect is particularly clear on day 5, where the intensity rapidly drops off in all three cases. On day 8, the rate of intensity reduction is lower, as cells have ventured onto all regions of the substrate, albeit at a lower density for higher ${\delta }_{{\kappa }_{2} < 0}$. The spatial nucleus distribution maps (Fig. 2f, g) can be quantified in a similar way by plotting the nucleus density (normalized by ${\delta }_{{\kappa }_{2} < 0}$ area) *vs*. ${\delta }_{{\kappa }_{2} < 0}$, showing a similar trend (Fig. 3b). The rapid increase in the nucleus density at high ${\delta }_{{\kappa }_{2} < 0}$, which is primarily apparent for the day 8 data on the spherical substrates, is attributed to the relatively low number of regions with such high values of ${\delta }_{{\kappa }_{2} < 0}$, causing an artificial spike in the normalized nucleus density. Nevertheless, these data on the F-actin and nucleus distribution show that, at extended time scales, the cells can collectively conquer curvatures that are not initially attractive. In this regard, it is not the instantaneous curvature that governs cell patterning but the presence of a region with ${\kappa }_{2} \, < \,0$ in the vicinity. Though not of primary interest, we also explored how curvature magnitude affects cell organization. Therefore, we also fabricated cell culture chips with convex spherical and unduloid substrates scaled at 50%, 100%, and 200%. The 50%-scaled (spheres: 90 µm radius) and 200%-scaled (spheres: 360 µm radius) substrates present the cells with twice as high and twice as low radii of curvature compared to the default 100% substrates (spheres: 180 µm radius). We found that the regions with ${\kappa }_{2} \, > \,0$ on the 50%-scaled substrates were more clearly avoided by the cells, even at the longest time point, which was not the case for the 100 and 200% substrates. This indicates that the unfavorable curvatures are of too high magnitude in the 50%-scaled substrates to be conquered by the cells after 8 days.

To further explore the role of cell-substrate interactions, we repeated experiments on substrates made from tissue culture polystyrene (PS). Using a proven solvent-based approach (“Methods”), the structures were accurately molded in PS and subsequently oxygen plasma-treated to promote cell adhesion, as is typically done for tissue culture plastic^34,35^. Contrary to PDMS, where the low glass transition temperature enables fast hydrophobic recovery through migration of hydrophobic groups from the bulk material to the surface, the surface properties of plasma-treated PS have been shown to remain stable for extended periods of time (e.g. several weeks in contact with air)^34^. On the PS substrates, we found generally similar cellular organization as on the PDMS substrates, with cells preferentially located on regions with ${\kappa }_{2} \, < \, 0$ (such as the convex-to-planar transition) especially at the earlier time points, as indicated by F-actin and nucleus distributions (Supplementary Figs. 7–8). Some of the data suggests a relatively higher nucleus density on regions with ${\kappa }_{2} \, > \,0$ than on PDMS at the earlier time points (Supplementary Fig. 8c–d), although the general trend still indicates a decrease with increasing ${\delta }_{{\kappa }_{2} < 0}$. This difference could potentially be attributed to slight differences in the surface adhesion between the PS and FN-functionalized PDMS substrates. Indeed, on some of the convex PS substrates, we observed partial cell delamination at day 5 on regions with ${\kappa }_{2} \, > \,0$ (Supplementary Fig. 8b), indicating that the cells could cover at least part of this curved region to some extent. Nevertheless, most convex PS substrates exhibited similar coverage as the PDMS counterparts, with limited cell presence on ${\kappa }_{2} \, > \,0$ at day 5.

### Cells collectively bridge large regions with ${{{{{{\boldsymbol{\kappa }}}}}}}_{{{{{{\boldsymbol{2}}}}}}} \, < \, {{{{{\boldsymbol{0}}}}}}$

**Fig. 4:**
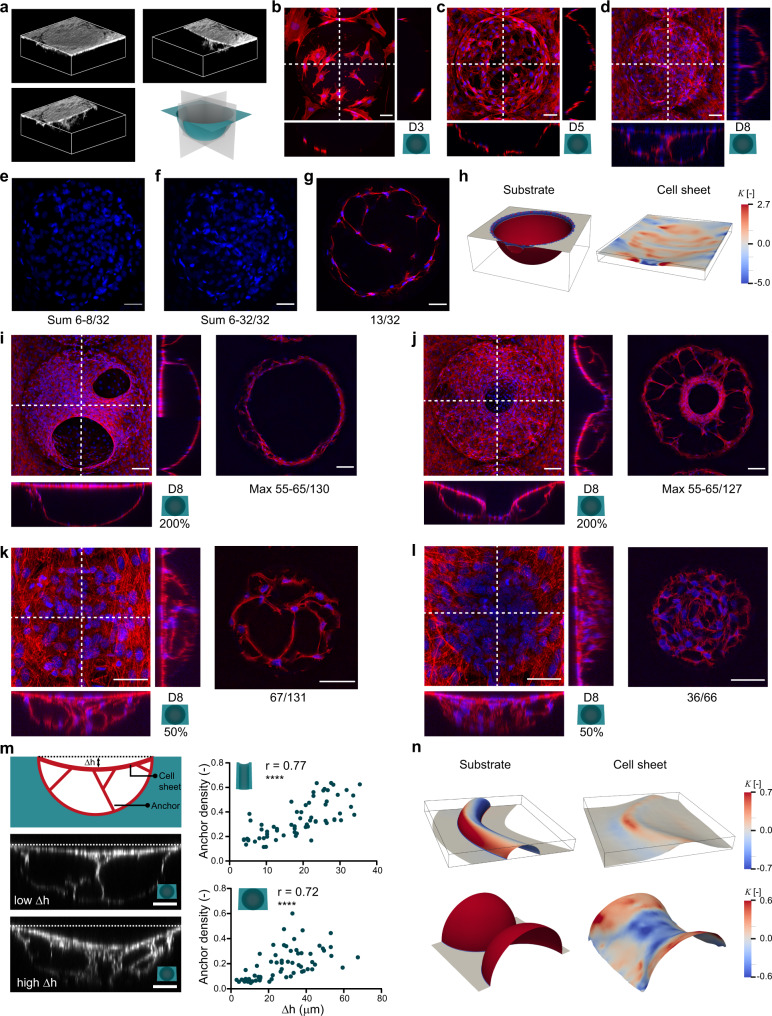
Curvature-induced cell bridging and formation of suspended cell sheets. **a** Cell sheet bridging over a concave spherical substrate. 3D reconstruction with cut-away views (see also Supplementary movie 2). **b**–**d** Representative maximum F-actin (red) and DNA (blue) intensity projections and cross-sectional views on spherical wells at days 3, 5, and 8, respectively. **e** Sum projection (DNA) of three slices at the top side in the z-stack from **d**. **f** The same as in **e**, but also including all slices underneath the cell sheet that bridges the concave well. **g** Single slice (13 of 32) of the z-stack from **d**, displaying a multicellular anchor in the spherical well (F-actin in red, DNA in blue). **h** Comparison of the area-normalized Gaussian curvature $\widetilde{K}$ of the concave spherical substrate (left) and the top surface of the tissue at day 8 (right). **i**, **j** Maximum intensity projections and cross-sectional views of F-actin (red) and DNA (blue) on large concave spherical wells at 200% scaling, showing two instances of incomplete coverage of the cell sheet. The inset figures on the right are maximum intensity projections of a few slices through the z-stack. **k**, **l** Similar as for **i**, **j**, but now for concave spherical wells at 50% scaling. **m** Quantification of cell sheet displacement (Δh) in relation to the anchor density for concave cylindrical substrates (top graph) and concave spherical substrates (bottom graph). The *r*-value indicates the Spearman’s correlation coefficient; ****p < 0.0001 (two-tailed). The data from the cells treated with TGF-β and blebbistatin are also included in these graphs. Data from three independent experiments. **n** Area-normalized Gaussian curvature coloring of the concave sinusoidal and spherical substrates (left) and the segmented tissue surfaces (right). All scale bars are 50 μm, except the ones in **i**, **j**), which are 100 μm. Source data are provided as a Source Data file.

Closer inspection of the image stacks on the concave substrates revealed that cells do not uniformly fill the concavities, but form suspended cell sheets that span the entire curved region while remaining anchored to the substrate through cell bridges (Fig. 4a and Supplementary movies 2–5). As demonstrated on a spherical substrate, the establishment of a suspended sheet begins with the individual exploration and spreading of cells in the spherical well (Fig. 4b). After 5 days, the cell density is high enough for the cells to link up and exert tensile forces to each other, enabling them to lift off the substrate and form bridges (Fig. 4c). After 8 days, the bridging cells have coalesced into sheets that span the entire concavity, while cell bridges underneath the sheet form anchors to the substrate (Fig. 4d, Supplementary Figs. 5–6). These phenomena were not exclusive to the spherical substrates but were observed in all concave substrates after 8 days, across the entire substrate length (Supplementary Fig. 4a). Curvature-induced cell bridging has also been observed in individual mesenchymal stromal cells (MSCs)^8,36^, and cell sheet detachment has been reported in smooth muscle cells^37^ and cardiomyocytes^38^ seeded in microgrooves, though along shorter lengths than we have observed. Further investigation of the bridging on the concave spherical substrates on day 8 reveals that most cell nuclei are situated in the suspended cell sheet, which is monolayered (Fig. 4d–f). Relatively few cells are present underneath the monolayered sheet, and many of these cells form bridges between the cell sheet and the substrate. These bridges are generally multicellular and consist of highly stretched cells with long F-actin processes and compressed nuclei, resulting in relatively higher DAPI intensity (Fig. 4f, g). Despite many cells being suspended across the concavity, cells lining the surface of the concavity continued to be observed (cross-sectional views in Fig. 4d and Fig. 4i–l). This indicates that the cell-surface adhesion at the concavities is not compromised or degraded beyond the point that cells cannot adhere to the surface anymore. We observed the same patterns of cell organization on the micromolded PS substrates as on the fibronectin-functionalized PDMS substrates. Specifically, we also found cells to bridge the concave regions after 8 days, forming a suspended cell sheet that spans the concavity with anchoring bridges underneath (Supplementary Fig. 9). While others have observed individual cell lifting on poly(trimethylene carbonate) or hydroxyapatite^36,39^, our experiments robustly demonstrate large-scale cell bridging across a variety of concave shapes made from both PDMS and PS, indicating that this bridging behavior is likely generalizable to other materials as well.

The bridging behavior of the cells and the formation of a suspended cell sheet causes a drastic change in the overall geometric landscape, as evidenced by the differences in the Gaussian curvature spectrum between the original concave spherical substrate and a 3D reconstruction of the detached cell sheet spanning that substrate (Fig. 4h). As such, new cells that would migrate in this environment experience different geometric cues than the original cells populating the curved substrates. To explore how the size of the hemispherical concavity affects the bridging behavior, we also cultured cells for 8 days on concave upscaled (200% scale, 720 µm diameter) and downscaled (50% scale, 180 µm diameter) spherical substrates. Cell bridging was also observed on the 200%-scaled substrates, but only partial cell sheets were formed by day 8. These cell sheets exhibited circular holes and did not fully cover the large hemispherical concavities (Fig. 4i, j). We observed circumferentially oriented stress fibers with relatively high F-actin expression at the edges of the holes, which is indicative of the mechanical tension that exists in the suspended sheet (Supplementary Fig. 21). Furthermore, we also found some evidence of stretched radial stress fibers that bridge the hemispherical substrate and the circular hole of the incomplete cell sheet (Fig. 4j), in a manner that is reminiscent of wound closure, where mechanical forces also play a crucial role. On the 50%-scaled substrate, we observed full coverage of the concave hemispheres on day 8 (Fig. 4k, l). Moreover, these smaller concavities appeared more crowded with cells than the 100 and 200%-scaled substrates, and the distinction between a detached cell sheet and sparse cell bridges underneath was not as clear. Instead, cells seemed to be randomly dispersed throughout the concave well, forming some stretched actin processes, but not to the same extent as on the larger concavities.

When evaluating cell sheet suspension across specimens, we observed a variation in the vertical sheet displacement ($\Delta h$ in Fig. 4m). To discern whether this was related to the presence of anchors, we calculated $\Delta h$ and the anchor density below the sheet for standard experiments on spheres and cylinders, as well as for the experiments with up- or downregulated contractility (see Section “Perturbation of cell contractility and osteogenic differentiation affects curvature guidance”). As expected, we observed a positive correlation between $\Delta h$ and the anchor density (Spearman’s $r=0.77$ for the cylinders and Spearman’s $r=0.72$ for the spheres). We also looked for evidence of cell sheet detachment on the convex curved substrates. We observed cell sheet detachment at the convex-to-planar transition (${\kappa }_{2} \, < \,0$) on all convex structures, though most notably on the sinusoidal cylinder (Fig. 4n and Supplementary Fig. 19). Interestingly, we found that sheet suspension is much more pronounced at the concave side of a substrate bend, exhibiting a detached sheet that departs from the top of the substrate and is suspended over a distance that is 4 times longer than at the convex side of the bend (Fig. 4n and Supplementary movie 5). At the concave side, the cells collectively sense the concavity of the transition region (${\kappa }_{2} \, < \,0$) as well as the overall concavity of the substrate (${\kappa }_{1} \, < \,0$). The combination of these curvatures ($K \, > \,0,{H} \, < \,0$) appears to stimulate the formation of a substantial detached cell sheet, suspended over a large region of the planar surroundings. A similar behavior was observed at the sharp curvature transitions on the convex hemispherical substrate, where cells were found to bridge both the convex-to-planar transition and the narrow region between both hemispheres, resulting in an overall cell sheet geometry with a smooth saddle-shaped neck region that approximates that of a catenoid. Indeed, similar to the observations on concave hemispheres, the formation of a suspended cell sheet on convex substrates results in a general smoothing of the geometric landscape, where the cells bridge sharp curvature transitions and reduce the overall curvature spectrum. This large-scale cell bridging across convex-to-planar transitions was also present on the convex PS substrates, exhibiting a very similar organization as on the PDMS substrates (Supplementary Fig. 7d–f).

### Curvature induces collective stress fiber orientation

**Fig. 5:**
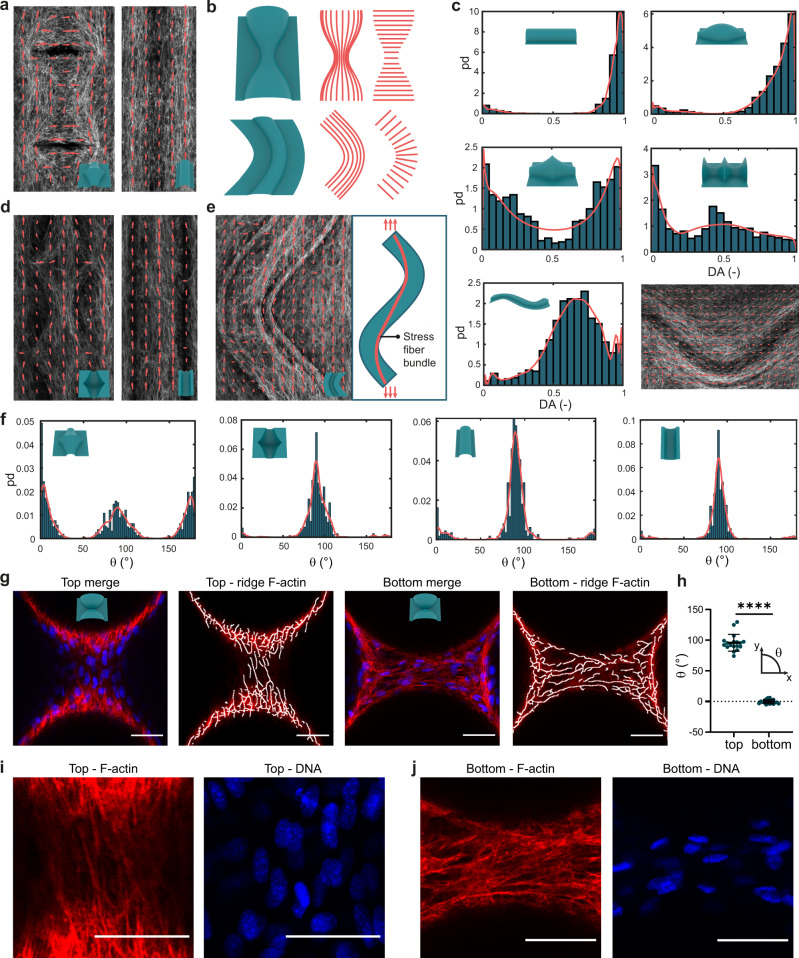
Collective stress fiber orientation on curved substrates. **a** Local dominant stress fiber orientations on convex substrates, computed for superpixels containing 80 × 80 pixels on maximum intensity projections of F-actin. **b** Illustrations of the principal directions on some portions of the convex unduloid (top) and convex sinusoidal cylinder (bottom). **c** Probability density distributions (PD) of the degree of alignment (DA) of the SF with respect to the first principal direction (corresponding to ${\kappa }_{2}$) on the convex substrates (DA = 1 means perfect alignment with the first principal direction). Data are obtained from all the specimens (*n* ≥ 3), using superpixels of 80 × 80 pixels. The red lines represent Epanechnikov kernel density estimates. The inset figure on the bottom right displays the local orientation of SF on the convex sinusoidal cylinder. **d** Orientation of SF on the concave pseudospheres (left) and concave cylinder (right), computed for superpixels of 80 × 80 pixels. **e** Orientation of SF on the concave sinusoidal cylinder. The right panel schematically illustrates the presence of the central SF bundles that display a lower-amplitude wave. **f** PD distributions of the local orientation of SF (with respect to the horizontal axis) on the convex and concave pseudospheres and cylinders. The data were obtained from all the specimens, using superpixels of 80 × 80 pixels. The red lines represent the Epanechnikov kernel density estimates. **g** SF subpopulations on the convex spheres (top: longitudinal orientation (90°), bottom: horizontal orientation(0°)). The first and third panel show merged F-actin and DAPI images. The second and fourth panel show F-actin SFs overlaid with algorithmically detected ridges (ridge detection in Fiji). **h** The computed SF orientations of the subpopulations on the convex spheres. *n* = 18 different examined regions over three independent experiments. Data are presented as mean values +/− SD. Paired two-tailed *t*-tests: ****p < 0.0001. **i**, **j** Higher magnification images (F-actin and DNA) of the central regions in **g**). All data at day 8. All scale bars are 50 µm. Source data are provided as a Source Data file.

We next asked how curvature affects the collective orientation of stress fibers (SF) on our substrates (Fig. 5a). While curvature-induced orientation has been observed in individual cells^13,40^, cells in monolayers and in developing tissues have shown to cooperatively sense weaker curvature fields^15,31^. From a differential geometric perspective, it is interesting to compare the orientation of SF to the principal directions of the curved substrates, which are the directions along which ${\kappa }_{1}$ and ${\kappa }_{2}$ occur (Fig. 5b and Supplementary Fig. 10). The cells with pronounced SF have been previously found to align along the direction of minimum principal curvature (${{{{{{\bf{P}}}}}}}_{{{{{{{\boldsymbol{\kappa }}}}}}}_{{{{{{\bf{2}}}}}}}}$), which is often attributed to the tendency to minimize the bending energy of SF^13,20^. We calculated SF orientation using a Fourier-based approach, focusing on the curved regions of the substrates (Supplementary Figs. 10–12), and we quantified the degree of alignment (DA) between the SF and ${P}_{{\kappa }_{2}}$ on the convex substrates. The spherical substrates were excluded, since the principal directions are not defined for spheres (${\kappa }_{1}={\kappa }_{2}$). The strongest DA was observed on the cylinders, showing the collective orientation of SF along the zero-curvature direction (${\kappa }_{2}=0$) (Fig. 5c). While the cells were also found to align well with ${{{{{{\bf{P}}}}}}}_{{{{{{{\boldsymbol{\kappa }}}}}}}_{{{{{{\bf{2}}}}}}}}$ on the unduloid, this was not found to be the case for the pseudospheres and catenoids. On those saddle-shaped substrates, the DA distributions indicate a lower overall alignment with ${{{{{{\bf{P}}}}}}}_{{{{{{{\boldsymbol{\kappa }}}}}}}_{{{{{{\bf{2}}}}}}}}$, yet the peaks at DA = 0 suggest some alignment with ${{{{{{\bf{P}}}}}}}_{{{{{{{\boldsymbol{\kappa }}}}}}}_{{{{{{\bf{1}}}}}}}}$ (${{{{{{\bf{P}}}}}}}_{{{{{{{\boldsymbol{\kappa }}}}}}}_{{{{{{\bf{1}}}}}}}}$ and ${{{{{{\bf{P}}}}}}}_{{{{{{{\boldsymbol{\kappa }}}}}}}_{{{{{{\bf{2}}}}}}}}$ are orthogonal). Interestingly, the cells on the sinusoidal-cylinder were found to collectively align, yet with some deviation from the principal direction ${{{{{{\bf{P}}}}}}}_{{{{{{{\boldsymbol{\kappa }}}}}}}_{{{{{{\bf{2}}}}}}}}$ (DA $\approx$ 0.7). We attributed this deviation to a bypassing effect, whereby SF follow the sinusoidal orientation of the substrate to some extent, but exhibit a collective resistance to change their orientation in response to the alternating curvatures. On the concave substrates, where the cells coalesced in detached sheets, we also observed the collective alignment of SF. In the channel-like substrates (i.e., cylinder, unduloid, catenoids, and pseudospheres), we found a strong longitudinal preference, typically exemplified by the presence of a central SF bundle (Fig. 5d, f). This longitudinal preference was attributed to a confinement effect^41^, where cell crowding in the detached sheet induces collective alignment after 8 days. Indeed, we did not observe longitudinal alignment in the concavities after 5 days, when cells were forming randomly oriented local bridges (Supplementary Fig. 4c). Considering the concave sinusoidal cylinder, we observed SF bundles that trace a lower-amplitude sinusoidal path than the original substrate (Fig. 5e). This path is reminiscent of the shape that a tensioned string confined to a sinusoidal channel would adopt, implying a role for actomyosin contractility in the collective orientation of SF.

Finally, we investigated the SF orientation in the regions where superimposed layers of cells were observed, such as on the saddle-shaped region of the convex unduloid or at the saddle-shaped transition between convex spheres. Interestingly, we found distinct SF subpopulations with orthogonal orientations on both substrates (Fig. 5g–j, Supplementary Fig. 15, and Supplementary movies 7–8). In the lower focal planes, laterally oriented SF were observed (x-direction), while the top focal planes displayed longitudinally oriented SF (y-direction, Fig. 5g). In a different study, orthogonally oriented SF have been observed within individual cells on saddle shapes^19^. It was postulated that SF above the nucleus preferentially align in the concave direction to minimize bending, while SF below the nucleus align in the convex direction to support cell migration. Based on these observations, we hypothesize that lower SFs align in the convex direction when cells migrate onto the substrate from both sides and form mechanical connections. Once these regions have been conquered, new cells can align in the concave direction to minimize bending. The orthogonal alignment in the different focal planes is also reflected in the orientations of the cell nuclei, which follow the orientations of the SFs. In the top planes, the ellipsoidal-shaped nuclei are oriented in y-direction, while the bottom planes exhibit nuclei aligned in the x-direction (Fig. 5i, j and Supplementary Fig. 15). The bottom planes also typically featured several cells with highly stretched SF and with nuclei of relatively high aspect ratio, which could be indicative of a higher mechanical tension in the bottom region as compared to the (more relaxed) top region.

### Expression of osteoblast differentiation marker on curved substrates

**Fig. 6:**
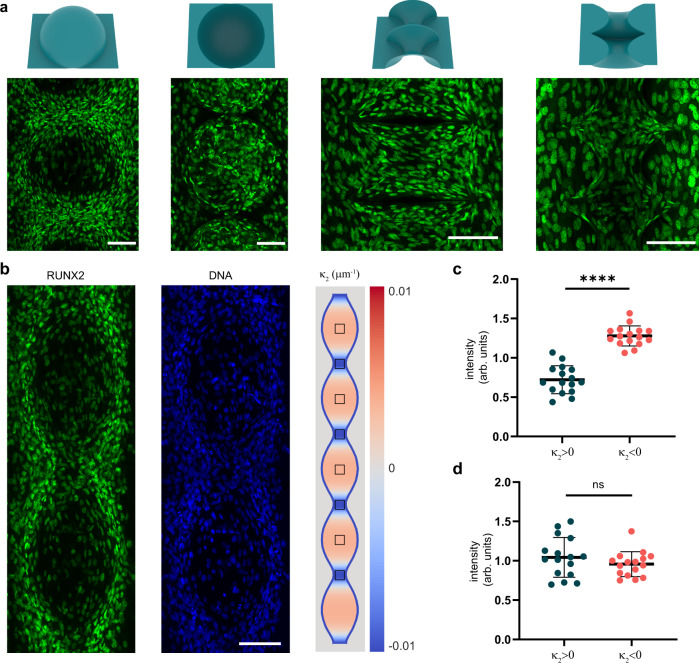
RUNX2 expression on curved substrates. **a** Representative maximum intensity projections of RUNX2 expression at day 8 on the convex sphere, concave sphere, convex catenoid, and concave catenoid substrates. **b** Maximum intensity projection of RUNX2 and DNA (DAPI) expression on a convex unduloid substrate. On the right side of the panel, a curvature map of ${\kappa }_{2}$ of the convex unduloid is shown. The squares (110 × 110 pixels) indicate the regions of interest (ROI) used in the subsequent analysis. **c**, **d** RUNX2 (**c**) and DAPI (**d**) mean ROI intensity versus the sign of ${\kappa }_{2}$, calculated using the approach shown in Supplementary Fig. 16 (every datapoint corresponds to a single ROI, *n* = 16 different ROIs from four independent experiments). The mean intensity of the ROI was normalized with respect to the mean intensity of all ROIs within the same image. Data are presented as mean values +/− SD. Unpaired two-tailed *t*-test, ****p < 0.0001, ns: not significant. All scale bars are 100 µm. Source data are provided as a Source Data file.

While the primary interest of this study was in spatial cell organization and orientation in the curved landscapes, our use of a preosteoblast cell line raised the question whether we could observe a potential curvature-dependent differential expression of an osteoblast differentiation marker. Therefore, we stained the cells on day 8 for runt-related transcription factor 2 (RUNX2), which is a well-known hallmark for osteoblast differentiation. We observed clear RUNX2 expression in the cell nuclei on all curved substrates on day 8 in the control medium, both on the convex and the concave variants (Fig. 6a). On the convex spherical substrates, which present the cells with large regions with ${\kappa }_{2} \, > \,0$, we qualitatively observed relatively higher RUNX2 intensities in the saddle-shaped regions between both hemispherical caps, as opposed to on the center of the convex caps. Quantifying these differences, however, is complicated by the fact that the nuclei of the cells in those saddle-shaped regions appear to be laterally compressed by the high degree of stretch that many of these cells experience (see also Fig. 5j), which could affect the average RUNX2 fluorescence intensity detected in those nuclei. Therefore, we focused our quantification on the convex unduloid substrate, which presents regions of interest (ROIs) of ${\kappa }_{2} \, > \,0$ and ${\kappa }_{2} \, < \,0$ that are relatively parallel to the focal planes and do not exhibit such highly compressed nuclei (Fig. 6b and Supplementary Fig. 16). Comparing the mean RUNX2 intensities in both regions revealed consistently higher intensities for saddle-shaped (${\kappa }_{2} < 0$) regions as opposed to spherical (${\kappa }_{2} \, > \,0$) regions, while no significant difference in DAPI intensities was detected. Others have also observed a similar curvature-dependent differentiation effect in MSCs and it has been argued that this could be linked to curvature-mediated changes involved in actin-induced nuclear compression^20,36^. These observations warrant further investigation to uncover mechanistic insight, but already point towards a potential role for substrate curvature in the regulation of cell differentiation.

### Perturbation of cell contractility and osteogenic differentiation affects curvature guidance

**Fig. 7:**
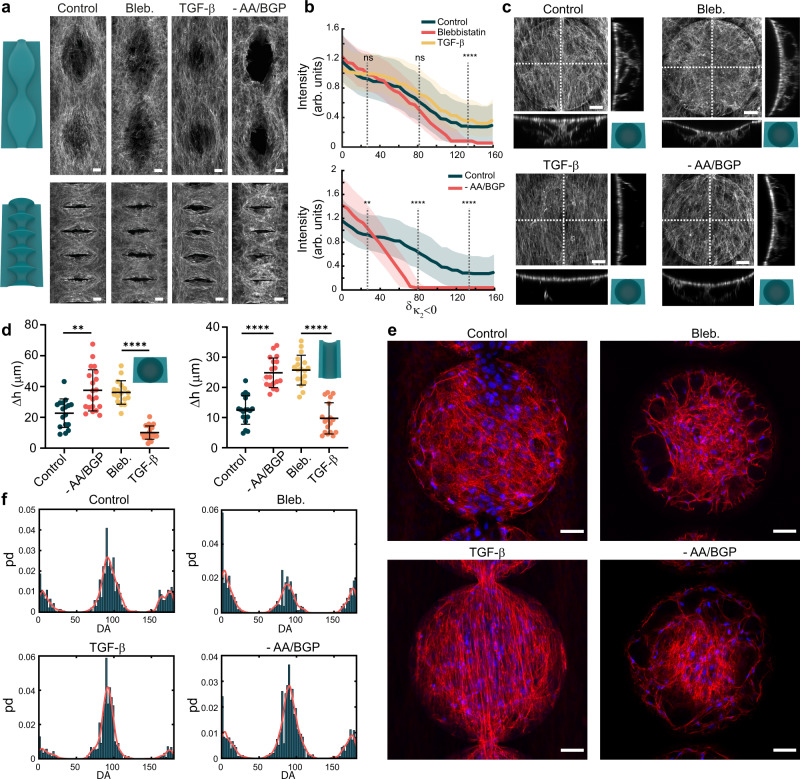
Effect of contractility and differentiation perturbation on the curvature-induced organization. **a** Representative maximum intensity projections (F-actin) on the convex unduloids (top) and catenoids (bottom), displaying the effects of contractility inhibition (Bleb.), contractility enhancement (TGF-β), and differentiation inhibition (-AA/BGP). **b** Intensity reduction as a function of ${\delta }_{{\kappa }_{2} < 0}$ for the convex spheres. The top chart demonstrates the effects of contractility perturbation (Kruskal–Wallis test: ****p < 0.0001). The bottom chart demonstrates the effects of differentiation inhibition (Two-tailed Mann–Whitney test: **p = 0.0057, ****p < 0.0001). The solid lines represent the median values while the shaded areas represent the interquartile range. **c** Effect of contractility perturbation and differentiation inhibition on cell sheet detachment over concave hemispheres at day 8. **d** Quantification of cell sheet displacement (Δh) due to contractility perturbation or differentiation inhibition. Data from multiple regions on three independent experiments. Data are presented as mean values +/− SD. Brown-Forsythe and Welch’s ANOVA with Dunnett’s T3 multiple comparisons: **p = 0.0017, ****p < 0.0001. **e** Merged F-actin (red) and DNA (blue) expression at day 8 in single slices from z-stacks obtained on concave spherical wells, showing differences in SF morphology and orientation for the different culture conditions. **f** PD of the SF orientation on maximum intensity projections of concave spheres. Data from three independent experiments. The red lines represent Epanechnikov kernel density estimates. All scale bars are 50 μm. Source data are provided as a Source Data file.

Previous studies on cell- and tissue-level organization in complex geometric environments have consistently pointed towards a role for cellular contractility as a regulator of curvature guidance. Therefore, we also explored the role of perturbed contractility on the multicellular organization in our complex curved landscapes. To downregulate contractility, we supplemented the medium with blebbistatin, a commonly used disruptor of actomyosin contractility through myosin-II inhibition. Motivated by previous studies, we enhanced contractility using the multifunctional cytokine transforming growth factor-β (TGF-β). This pleiotropic cytokine is involved in a variety of biological processes, including cell differentiation, migration, tissue homeostasis (e.g. bone), wound healing, and several pathologies^42,43^. We supplemented the culture medium with TGF-β3, one of the three isoforms, which functions along the same receptor signaling pathways as the other isoforms. While the TGF-β/SMAD signaling pathway is the canonical pathway, TGF-β is also involved in other signaling routes such as the Wnt and Notch pathways, as well as Rho GTPases. Specific to cellular contractility, extended TGF-β stimulation (>24 h) is known to induce stress fiber formation, in a process that depends on the SMAD pathway and the Cdc42 and RhoA GTPases^44^. In general, we observed that the cells with perturbed contractility patterned the curved substrates similarly to the unperturbed cases, yet exhibited more- or less-pronounced SF in response to TGF-β and blebbistatin, respectively (Fig. 7a and Supplementary Fig. 17). However, we found that the cells treated with blebbistatin did not cover the unfavorably curved regions (${\kappa }_{2} \, > \,0$) as well as unperturbed cells (Fig. 7b). Since cell contractility is the driving force behind cellular bridging, we explored the effect of perturbing contractility on the morphology of the detached cell sheets and anchoring bridges on the concave substrates (Fig. 7c and Supplementary movies 3–5). Enhancing contractility results in lower sheet displacement and fewer bridges as compared to the cases where contractility is inhibited (Fig. 7d), implying that higher cell contractility translates to a higher overall tension in the suspended sheets. When considering specific focal planes within the cell sheets of the different cases, we also observed different SF morphologies and orientations. As compared to the unperturbed case, the cells treated with blebbistatin are more dendritic-like and form many small anchoring bridges that adopt circular configurations, while TGF-β induces strong SF with a more pronounced longitudinal alignment (Fig. 7e, f). Moreover, TGF-β treatment also resulted in the ability to completely cover the smaller concavities with a single sheet (Supplementary Fig. 13), and offered a higher coherency in alignment of the SF (Supplementary Fig. 14). The effects of collectively enhanced contractility were also apparent on the convex substrates, where strong cell sheets were observed that bridge the underlying curved structures. On the spherical substrates, for example, the cells cultured under normal conditions form modest bridges in between the spheres, while contractility-enhanced cells form much stronger cell sheets that remain almost planar and seem to avoid the underlying curvature (Supplementary Fig. 19a). These results are in line with previous studies at the cell and tissue scales^20,31^, and underpin the important role of cell contractility as a driving force for the multicellular organization in varying curvature fields. Our observations of stronger cell sheets and more apparent SF formation due to TGF-β treatment are in line with previous studies that used TGF-β to enhance collective contractility. However, TGF-β is a multifunctional cytokine that does not directly target actomyosin contractility but affects many more cell functions, the complete set of which has not yet been uncovered. Therefore, we also briefly explored the effects of more directly upregulating contractility through the addition of calyculin-A (CalA). CalA inhibits the dephosphorylation of myosin by myosin-light-chain phosphatase, effectively enhancing myosin activity^45^. Treatment with 0.5 nM CalA did not result in strong cell sheets or SF formation as in the case of TGF-β but did lead to the formation of extremely stretched cell bridges, spanning longer distances and covering more cells than those observed in the other culture conditions (Supplementary Fig. 18). These observations warrant further investigations (e.g. further optimization of the CalA concentrations), yet indicate that directly targeting myosin action using CalA does not result in the same level of tissue-scale contractility enhancement and overall organization as is achieved by TGF-β addition.

The osteogenic culture medium that was used during our experiments from day 3 onwards contained ascorbic acid (AA) and β-glycerophosphate (BGP), which support the development of mineralized extracellular matrix (ECM) and promote osteoblast differentiation^46^. AA induces the secretion of type I collagen (Col1) in the ECM, while BGP works synergistically with AA and acts as a phosphate source for mineralization^46–48^. To study how cells would organize on the curved substrates in conditions that are less stimulating of osteoblast differentiation and ECM synthesis, we performed experiments with a culture medium deprived of AA and BGP^39^. While the cells cultured in this medium generally exhibited similar curvature-induced organization, we observed significantly lower degrees of cell coverage on unfavorably curved regions (${\kappa }_{2} \, > \,0$) (Fig. 7a, b and Supplementary Fig. 17). On the concave structures, we found that AA/BGP deprivation resulted in weaker cell sheets, displaying similar SF morphologies and $\Delta h$ as compared to the cells treated with blebbistatin (Fig. 7c–g). Interestingly, we observed that the cells cultured in non-osteogenic medium in some cases collectively pulled away from the concave bends of the convex sinusoidal cylinders, a phenomenon that was never observed in the cells cultured in osteogenic medium (Supplementary Fig. 19b). These results suggest that stimulating osteogenic differentiation and ECM development, induced by the osteogenic supplements in the medium, enhances the ability of the cells to collectively cover unfavorable curvatures (${\kappa }_{2} > 0$) within our complex landscapes.

**Fig. 8:**
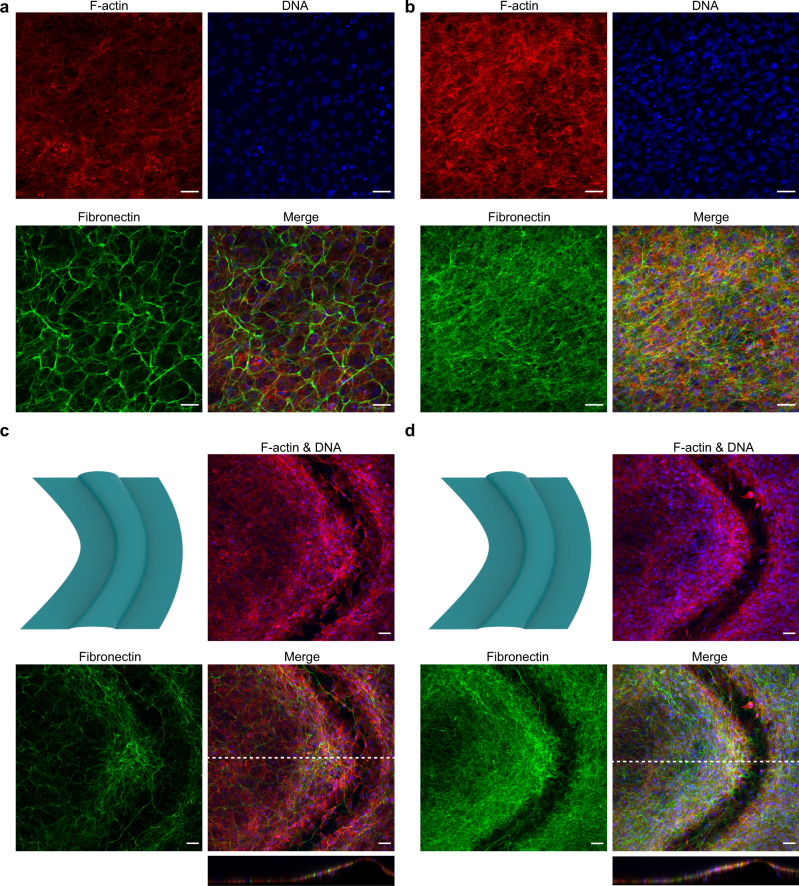
The effect of osteogenic supplement deprivation on early ECM development. **a**, **b** Representative fluorescent staining of F-actin (red), DNA (blue), and fibronectin (green) on a flat region after 8 days, in medium deprived of osteogenic supplements (-AA/BGP) (**a**) and in osteogenic medium (**b**). **c**, **d** Same as for **a**, **b**, but on a convex wavy substrate. The bottom insets are the cross-sectional views of the merged images. All scale bars represent 50 µm.

To further explore the effects of osteogenic supplements on the ECM development, we stained for mouse fibronectin (FN) after 8 days of culture in both supplement-rich and supplement-free conditions (Fig. 8). Previous studies have also explored the FN network deposited by MC3T3-E1 cells grown in geometrically structured environments^39^. Indeed, the cells deprived of AA and BGP exhibited a much sparser network of FN fibrils than those grown in an osteogenic medium, which displayed a dense meshwork. This difference in FN network assembly is clear both on flat regions as well as within the curved landscapes, the latter being exemplified by the substantially denser meshwork in the concave bend of the convex wavy substrate (Fig. 8d). The formation of a dense FN network likely acts as a supporting scaffold to the cells and facilitates their growth on unfavorably curved regions. In vivo, FN fibrillogenesis is also a necessary prerequisite for the assembly of collagen fibers in the ECM, and Col-I fibers have been shown to preferentially co-localize with relaxed FN fibrils^49^. We stained for Col-I (Supplementary Fig. 20) and explored second harmonic imaging, but could not find evidence of mature Col-I fibers in the ECM, which we attribute to the relatively low seeding density and the relatively short culture time. Nevertheless, the clear differences in FN fibril network in both culture conditions highlight the importance of ECM development during curvature guidance and for the ability of cells to cover unfavorably curved regions. Previous studies have also found that AA deprivation could affect the proliferation of MC3T3-E1 cells, in a process that has been linked to collagen synthesis, and osteogenic supplements have been found to affect cell contractility in hMSCs^50,51^. This could also contribute to the altered curvature-guided cell organization observed under non-osteogenic conditions, and warrants further investigation.

## Discussion

We have demonstrated the collective organization of preosteoblasts in cell-scale, varying-curvature landscapes. By designing mathematically defined surface families with controlled curvature variations and by leveraging high-resolution fabrication, we micro-engineered substrates that cover a wide range of Euclidean, hyperbolic, and elliptical geometries. We found that cells preferentially pattern regions with at least one concave direction (i.e., regions where the minimum principal curvature is negative, ${\kappa }_{2} \, < \,0$), while curved regions with ${\kappa }_{2}\ge 0$ are generally avoided. Previous studies have also reported preferences for local concavities, which has been attributed to a more relaxed stress configuration in the concavities^20,30,36^. However, those observations were typically made from a 2D perspective or without addressing the formal mathematical framework of surface curvature. Specifically, curvature guidance has often been studied by considering cell behavior on convex or concave substrates, typically spheres or cylinders, without addressing the specific mean or Gaussian curvatures. Moreover, hyperbolic substrates, containing both concave and convex directions, have received little attention until recently^14,31^, despite their high physiological relevance^8^. Our results show that one concave direction in a specific region is sufficient for cells to preferentially cover that region. This includes not only all hyperbolic geometries ($K \, < \,0$) but also elliptical ($K \, > \,0$) and Euclidean ($K=0$) regions where the mean curvature is negative. The convex-to-planar transition regions also belong to this category, and our results confirm that these have a clear influence on spatiotemporal cell organization, despite being often ignored in previous studies. These observations warrant further investigation into the presence of localized curvature peaks in relatively planar environments, but also into the effects of fully smooth environments devoid of such transition regions. Nevertheless, our data indicates that there is no single mean or Gaussian curvature that could be pinpointed as ideal for cell patterning, but rather a spectrum of shapes where ${\kappa }_{2} \, < \,0$. Despite a general preference for ${\kappa }_{2} \, < \,0$, we found that cells can eventually conquer unfavorably curved regions (${\kappa }_{2} \, > \,0$) through cooperative action, provided that the distance to favorably curved regions is not too large. However, this ability to venture onto curved regions with ${\kappa }_{2}\ge 0$ reduces when cell contractility or ECM production is impaired.

Our widespread observations of cell bridging and the formation of suspended cell sheets, including observations with blebbistatin and TGF-β, further illustrate the crucial role of cell contractility and the ability of the cells to collectively override the geometrical cues imposed by the substrate. Studying cell bridging is relevant to unravel the biophysics of force-induced cytoskeletal organization, but also to understand how cells migrate and organize within complex environments that present only discrete cell attachment points, such as adhesive fibers within the ECM. Cell bridging has previously been studied on 2D substrates decorated with adhesive and non-adhesive regions. For example, single cell experiments using mouse embryonic fibroblasts have revealed that tension homeostasis of cell bridges is regulated by the combination of myosin IIA-mediated contractile treadmilling flow and myosin IIB-mediated cross-linking stabilization^52^. The contractile apparatus also plays a crucial role in the bridging and gap closure observed in cell monolayers on 2D substrates with patterned non-adhesive domains^53,54^, and the 2D curvature of these domains (convex versus concave) was recently identified as an additional regulator in the formation of actin cables^55^. In 3D, individual hMSCs on concave spherical substrates formed detached cell bridges that are similar to 2D cell bridges by virtue of their discrete anchoring points to the substrate, their concave cell edges, and their highly stretched morphology^36^. Collective, contractility-dependent cell detachment has also been observed in smooth muscle cells and cardiomyocytes seeded in straight, constant-curvature mesoscale channels, but not in endothelial cells due to their lower contractile force generation^37,38^. Our results demonstrate the formation of multicellular bridging in preosteoblasts across a variety of different geometries, not only across large-scale concave structures, but also across local concavities around predominantly convex substrates. Our experiments show that these bridges can mature into fully suspended, monolayered cell sheets, often with longitudinal F-actin alignment in the channel direction while multicellular anchoring bridges to the substrate are found underneath the detached sheets. Our experiments with perturbed contractility are in line with previous results on 2D and 3D cell bridging^36,53,54^, confirming the central role of cellular contractility in bridge formation. The upregulation of overall contractility through TGF-β, an approach which has been used by others in similar investigations, resulted in more pronounced SFs with more coherent alignment and stronger overall cell sheets. Interestingly, we did not find the same results in our experiments aimed at directly upregulating myosin through CalA. In future studies, it will be interesting to study, in more detail, how the individual components of the collective force generation process, such as cell-cell attachment, myosin activity, or actin dynamics contribute to bridging and cell sheet detachment. Nevertheless, our data indicates that 3D extracellular geometry might not be a separate mechanobiological cue per se but cooperates with cell-generated forces to shape tissues. The precise intricacies of this interplay between geometry and tissue forces remain to be uncovered in further studies, likely relying on specialized experimental techniques, such as 3D traction force microscopy, Förster resonance energy transfer imaging, or atomic force microscopy, and potentially aided by computational modeling. Such targeted mechanobiological investigations could also shed light on the way that large-scale curvature triggers specific mechanotransduction pathways, e.g., relating geometry-mediated nuclear compression to cellular differentiation.

An important parameter governing these bridging and detachment phenomena is the cell adhesion to the substrate. Our substrates were functionalized by adsorption of fibronectin (FN), an ECM protein, which is commonly used to enhance cell adhesion to PDMS. Individual cell lifting has previously been observed on other substrate materials, such as poly(trimethylene carbonate) and hydroxyapatite^36,39^. In another study, cell sheets were found to detach from concave cylindrical channels with adsorbed protein coatings by rupturing the protein-substrate interface^37^. It was found that increasing the adhesion strength by covalently bonding the protein to the substrate could prevent detachment at sufficiently low curvatures and contractility, but in many cases still resulted in detachment. However, in those cases of detachment, the rupture plane was found to be shifted to the integrin-protein interface. Our results demonstrate large-scale multicellular bridging on two different materials (FN-functionalized PDMS and plasma-treated PS), not only across the large concave wells but also at the convex-to-planar transitions, but with small differences in time-dependent cell nucleus density on convex structures. This points to a competition between cell-surface adhesion and geometry-mediated cell detachment due to cellular contractility that is likely dependent on the choice of substrate material. For example, we found some indications of partial cell delamination on some convex PS substrates (Supplementary Fig. 8b) which were not apparent on PDMS substrates, suggesting the potential presence of material-related differences, though the general observations between the two material systems were similar. In future studies, it would be interesting to explore the interplay of these three factors further within the context of our complex curvature fields.

Our results on the curvature-induced collective orientation of SF are in line with the biophysical arguments that SF-dominated cells align in directions that minimize SF bending^13,21^. Indeed, we find that cells on convex cylinders or unduloids align well with the direction of minimum principal curvature. On the convex hyperbolic substrates (where $H \, > \, 0$), however, we found that cells show less uniform alignment, and that a substantial portion of SF align along a locally convex direction. Moreover, we observed orthogonally oriented SF subpopulations on some local saddles and confinement-induced longitudinal orientation of SF in detached cell sheets. In general, we concluded that substrate curvature, indeed, affects SF orientation, but that cell interactions, mediated by contractility and ECM production, result in a collective resistance to local variations in the underlying curvature.

Taken together, our results underpin the importance of local, cell-scale geometrical cues on the emergent organization of bone-like cells in complex and varying-curvature environments. In particular, these findings emphasize the role of multicellular cooperation, enabling cells to conquer unfavorably curved regions or alter their local environment through collective bridging. However, cells are typically exposed to several other biophysical cues in vivo, such as stiffness gradients^56^ or nanotopographies^6^. In this regard, it would be interesting to explore the multicellular cell organization in a tailored multi-cue environment to unravel the dominant cues and potential crosstalk^14^. Our results could ultimately inspire the design of tissue engineering scaffolds^10^. Based on our findings, one could argue that scaffolds with substantial regions with ${\kappa }_{2} \, < \,0$ are preferred. In scaffolds based on cylindrical strut networks, which have often been proposed^57–60^, such regions occur at the intersections of struts. Indeed, tissue formation has been found to initiate from those locations in vivo^61^. Alternatively, one could consider hyperbolic sheet-based scaffolds, such as those based on triply periodic minimal surfaces (TPMS), which have ${\kappa }_{2}\le 0$ at every point. However, developing geometrically optimized scaffold designs requires further investigation into the intricacies of cell-geometry interaction, likely involving computational studies that take geometry explicitly into consideration^62^. Nevertheless, fueled by rapid advances in high-resolution free-form fabrication, we anticipate exciting avenues for geometric control of cells and tissues, relying on surface curvature as the language of shape.

## Methods

### Design of curved substrates

The curved substrates were designed in Matlab (Matlab 2018b or 2020a, Mathworks, Natick, MA, USA) and SolidWorks (Dassault Systèmes, Vélizy-Villacoublay, France). For the axisymmetric substrates (i.e., unduloid, catenoid, sphere, pseudosphere, and cylinder), the generatrix curves were generated in Matlab using the parametrizations provided in Supplementary Note 1. The curves were then imported into SolidWorks and π-revolutions around the central axis were generated, resulting in convex half-surfaces of revolution. Next, end-caps and a rectangular bottom layer (with 20 µm thickness) were added to convert the surfaces to printable solids. The sinusoidal cylinder substrate was directly designed in SolidWorks, by sweeping a hemi-circular cross-section along a sinusoidal guiding curve. The corresponding substrate designs were exported in the STL format, to prepare them for the printing process.

### Fabrication and functionalization of PDMS substrates

Mold masters were fabricated using a two-photon lithography 3D printer (Nanoscribe GT2, Nanoscribe GmbH, Karlsruhe, Germany). A silicon substrate was cleaned with isopropanol (IPA, Merck KGaA, Darmstadt, Germany) and was treated with oxygen plasma (Femto, Diener electronic GmbH + Co. KG, Ebhausen, Germany) to improve its adhesion. A droplet of IP-S acrylate-based resin (Nanoscribe GmbH) was drop cast onto the substrate. Four sets of six convex master substrates were 3D printed on the silicon substrate using a 25× objective (NA = 0.8), 0.5 µm hatching, 0.5 µm slicing, 50 mW nominal laser power, and 50 mm/s scanning speed. To save time, an internal support scaffold was written instead of a solid block. The structures were developed in propylene glycol methyl ether acetate (PGMEA, Merck KGaA, Darmstadt, Germany) for 25 min followed by 5 min of treatment with isopropyl alcohol (IPA) and blow drying using a filtered air gun.

To perform the molding, the master was first placed in a vacuum desiccator beside a glass petri dish containing a droplet of trichloro(1H,1H,2H,2H-perfluorooctyl)-silane (Merck KGaA, Darmstadt, Germany), which subsequently coated the surface of the master with a hydrophobic layer allowing easy future peeling of the polydimethylsiloxane (PDMS) copy. PDMS (Sylgard 184, Dow Inc., Midland, MI, U.S.A.) mixed with the curing agent at a weight ratio of 10:1 was mixed thoroughly, drop-cast on the master, desiccated in vacuum for 30 min to remove any air bubbles, and cured in an oven at a temperature of 40 °C for 16 h. The resulting copy was cut out by scalpel and gently peeled off the master. Typically, 7–10 PDMS copies were made with a single master without loss of fidelity. This single-step molding process resulted in specimens with concave curved substrates (imprints in the PDMS). To obtain the convex counterparts (protrusions), a double molding step was performed. To this end, a PDMS copy originating from the single-molding operation was thermally aged in an oven at 100 °C for 48 h^63^. This PDMS substrate was consequently used as a new mold for the second molding stage, which was performed in the same way as before. After curing, the second PDMS could easily be peeled off the PDMS mold, without loss of fidelity. The quality of the single-step and two-step PDMS molding processes was verified using a scanning electron microscope (SEM, JSM-IT100LA, JEOL, Tokyo, Japan) with a beam energy of 10 kV and a working distance of 12 mm. Prior to SEM imaging, the PDMS substrates were gold sputtered (layer thickness of 5 ± 2 nm) to enhance conductivity. In addition, the quality of the replica molding procedures was confirmed through laser confocal scanning (Keyence VK-X 3D scanner, Keyence, Osaka, Japan) using a 20× magnification lens.

In preparation for cell seeding, circular specimens of 8 mm diameter were punched from the PDMS copies. Next, the specimens were sterilized inside an oven at 110 °C for 1 h. To reduce the inherent hydrophobicity of PDMS and facilitate substrate wetting, the specimens were subsequently treated with oxygen plasma (Femto, Diener electronic GmbH + Co. KG, Ebhausen, Germany) for 3 min. Then, the PDMS specimens were transferred to a 48-well plate, washed twice with 10 × PBS, submerged in a solution of 50 μg/ml bovine fibronectin (Sigma-Aldrich, St. Louis, MO, USA), and incubated at 37 °C and 5% CO_2_ for 30 min to uniformly functionalize the PDMS surface (Supplementary Fig. 22) and promote cell adhesion. After the removal of the fibronectin solution, the specimens were thoroughly washed with 10 × PBS.

### Fabrication of PS substrates

PS substrates were fabricated according to a previously published solvent-based molding method^34^. Briefly, tissue culture polystyrene petri dishes (Corning Falcon) were broken into small pieces and dissolved at 25 wt% in γ-butyrolactone (GBL) at room temperature while continuously stirring for several hours. The PS solution was then cast onto a PDMS replica and placed on a hotplate at 90 °C for 12 h, followed by 3 h at 120 °C to evaporate the solvent. Upon cooling, the PDMS mold was peeled from the solidified PS, revealing accurately molded replicas of the curved substrates on the PS base. Finally, the PS sample was cut and trimmed using a rotary tool. Prior to cell culture, the PS samples were treated with oxygen plasma for 1.5 min.

### Cell seeding and culture

Prior to cell seeding, murine preosteoblasts (MC3T3-E1, Sigma-Aldrich, St. Louis, MO, USA or ATCC, CRL-2593 (Supplementary Figs. 7–9)) were cultured for 7 days in minimum essential medium (α-MEM, Sigma-Aldrich) with the addition of 10% fetal bovine serum and 1% penicillin-streptomycin (both from Thermo Fischer Scientific, Waltham, MA, USA). The medium was refreshed every 2–3 days. In all the experiments, ~5 × 10^3^ cells were seeded on the fibronectin-coated PDMS specimens in 250 μl culture medium, which were then cultured for up to 8 days at 37 °C and 5% CO_2_ with the medium being refreshed every 2–3 days. To induce osteogenic differentiation, the culture medium was supplemented with 4 mM β-glycerophosphate and 50 μg/ml ascorbic acid (both from Sigma-Aldrich), starting from day 3. In the experiments where differentiation was inhibited, the cells were cultured in the standard culture medium throughout the entire duration of the experiments (i.e., without the addition of ascorbic acid or β-glycerophosphate). In the experiments with enhanced cell contractility, 1 ng/ml of TGF-β3 (Sigma-Aldrich) was added to the culture medium on day 3 and this concentration was maintained throughout the remainder of the experiments. To inhibit cell contractility, the culture medium was supplemented with 10 µM of blebbistatin (Sigma-Aldrich), which was also maintained throughout the remainder of the experiments.

### Fluorescent staining

Staining was performed at different time points (days 3, 5, and 8). The specimens were washed twice in 10× PBS and fixated in 4% formaldehyde/PBS for 15 minutes at room temperature. Next, the specimens were washed with 1× PBS and the cells were permeabilized in 0.5% Triton/PBS at 4 °C for 5 min, followed by incubation in 1% BSA/PBS at 37 °C for 5 min. To stain for F-actin, the specimens were incubated in 1% BSA/PBS with rhodamine phalloidin (1:1000, Thermo Fischer Scientific). Afterwards, the specimens were washed three times for 5 min with 0.5% Tween/PBS at room temperature, followed by washing for 5 min with 1 $\times$ PBS at room temperature. The specimens were subsequently mounted in a glass-bottom dish using a droplet of ProLong Gold antifade reagent with 4’,6-diamidino-2-phenylindole (DAPI, Thermo Fischer Scientific) to stain the chromatin cargo in the nuclei. The specimens that were also stained for RUNX2 or Collagen I followed a similar protocol, involving a first incubation step with anti-RUNX2 primary antibody (1:500, ab192256, Abcam, Cambridge, UK) or anti-Collagen I antibody (1:500, sab4500362 Sigma-Aldrich, St. Louis, MO, USA), respectively, followed by Tween/PBS washing and a second incubation step with Alexa Fluor 488 conjugated secondary antibody (1:500, Donkey anti-Rabbit IgG (H + L) A-21206, Thermo Fischer Scientific). Fibronectin staining was performed using an Alexa Fluor 488 conjugated anti-Fibronectin antibody (1:250, ab237286 Abcam, Cambridge, UK).

### Confocal imaging

Fluorescence confocal laser scanning microscopy (CLSM) was performed using a Nikon Eclipse Ti inverted confocal microscope (Nikon, Tokyo, Japan) with a Nikon Plan Apochromat λ 10× objective (0.45 NA) and the NIS-Elements software (version 4.51.01). The images were acquired using 2 or 3 laser lines with excitation wavelengths of 405 nm (DAPI), 488 nm (Alexa Fluor 488), and 561 nm (Rhodamine-phalloidin) with the detection windows set accordingly. Z-stacks were obtained at an *xy*-resolution of 0.60 × 0.60 µm and a z-spacing of 1 µm (for specific cases) to 5 µm (nominal cases). The acquisition in the different channels was performed sequentially to minimize inter-channel cross-talk. The images in Fig. 8 and Supplementary Figs. 7–9 were acquired using a Leica SP5 inverted confocal microscope (Leica Microsystems, Wetzlar, Germany) with a Leica HC PL APO 10× objective (0.40 NA) with Leica LAS AF software (version 2.7.3) under equivalent conditions as listed above.

### Preparation of stack projections and curvature maps

Maximum intensity projections were obtained from the image stacks using Fiji (version 1.53c)^64^. A custom image registration script in Matlab was used to select a rectangular region of interest (ROI), defined by the bottom layer of the curved substrates, and crop the image to the ROI. The resulting image was subsequently rotated to align the rectangular ROI in the vertical direction. To assess the relationship between the confocal image data and the curvature of the underlying substrate, curvature maps were created in Matlab using the parametrizations provided in Supplementary Note 1. The transition region between the curved substrates and the flat surroundings was assigned a radius of curvature of 15 µm to account for the local concavity that this narrow region presents. The curvature maps were defined as pixelated images, matching the resolution of the corresponding confocal images, enabling a pixel-by-pixel comparison of the confocal data and the curvature. The principal curvatures were non-dimensionalized by multiplying with the radius of the spherical substrate (leading to ${\kappa }_{1}={\kappa }_{2}=1$ for the convex spherical substrates).

### Actin frequency maps

To create the frequency maps of the actin images, several images belonging to the same experimental group were split into periodic units and superimposed in Matlab. First, the intensity was normalized with respect to the mean intensity of the image. All the images within the same group were then summed. Finally, the images were split into periodic units and summed again to create the final frequency map.

### Intensity quantification and distance maps

The actin maximum intensity projections were normalized with respect to the mean intensity and were converted to grayscale. Using custom Matlab scripts, every image was rasterized (downsampled) into a set of superpixels, where the intensity of each superpixel is the average of all the pixels it is composed of. Through this rasterized approach, the intensity variations are considered for local neighborhoods (better corresponding to the scale of the cells), rather than at the individual pixel level. The size of the superpixels is mentioned in the figure captions. The distance maps were created by binarizing the curvature maps of the minimum principal curvature (${\kappa }_{2}$). These binarized curvature maps were used to compute the Euclidean distance transform for which every pixel was assigned a value depending on its distance from the nearest point with a negative minimum principal curvature. To assign a higher weight to the regions with ${\kappa }_{2} \, > \,0$ than regions with ${\kappa }_{2}=0$ (corresponding to the observed relative preference of the cells for the latter as opposed to the former regions), the final distance map was defined as the average of two distance maps: a map representing the distance to points with ${\kappa }_{2}\le 0$ and a map with the distance to points with ${\kappa }_{2} \, < \, 0$.

### Nuclear centroid positions

Maximum intensity projections of the DAPI nuclear stainings were created and processed in Fiji. The images were manually thresholded and binarized. Subsequently, small holes were filled, the watershed algorithm was applied to separate touching nuclei, and the morphological opening operation was applied to remove small fragments that did not represent actual nuclei. Next, the centroid position of every nucleus was extracted and the list of positions was imported into Matlab for further processing. Incorrectly binarized fragments smaller than 15% of the median nuclear area were discarded. To create the frequency maps of the nuclei centroids, the centroid positions from at least three specimens (at least two in case of Supplementary Fig. 8) were combined, and the data was collapsed onto a single unit region. The resulting map of all nuclear centroid positions was then converted into a two-dimensional histogram of 100 × 100 bins, and subsequently convolved with a 3 × 3 Gaussian filter ($\sigma=1.5$), to yield the final frequency maps. For the data in Supplementary Fig. 8, two images in the dataset were manually processed to remove an artefact in the binarized images due to contamination in the DAPI channel, without disturbing the nuclear segmentation.

### Quantification of cell sheet detachment on concave spheres and cylinders

To quantify the amount of cell sheet displacement and the anchor density, the actin z-stacks were first cropped to a square ROI and then resliced from the top with 5 µm spacing in Fiji. Using custom scripts in Matlab, the middle image of the resliced stack was selected, the detached cell sheet was traced, and the maximum displacement of the sheet with respect to the endpoints of the sheet was quantified (Fig. 4m). The anchor density was quantified by masking and binarizing the region below the detached sheet in the resliced stacks. Consequently, the density of the actin voxels in the binarized image stack was determined. The 3D reconstructions of the confocal stacks were generated using the open-source Fiji plugin 3Dscript^65^.

### Cell sheet reconstruction and curvature estimation

To create the reconstructions of the detached cell sheets in Fig. 4h, n, orthogonal image stacks (cross-sectional view) of the F-actin z-stacks were first exported from Fiji and imported into the open-source 3D Slicer software (version 4.11). Next, the detached cell sheet, clearly visible in every image of the orthogonal stack, was segmented to create a 3D reconstruction, which was exported in.stl file format. The resulting high-density triangle mesh was further processed in the open-source Meshlab software (version 2021.07). First, the mesh was downsampled using quadric edge collapse decimation to reduce the overall file size and facilitate subsequent mesh processing steps, without sacrificing the overall mesh detail. In addition, self-intersections, non-manifold edges and vertices, and small holes in the mesh were removed to create a watertight mesh. Next, the mesh was imported into the open-source Meshmixer software (version 3.5) to clip off the side faces and only retain the top side of the 3D mesh. Then, the mesh was again imported in Meshlab and smoothed using three iterations of the “two-step” smoothing algorithm, which maintained the overall mesh geometry and smoothed out the irregularities that resulted from the segmentation process. The resulting mesh was then used for visualization and curvature estimation.

The Gaussian curvature of the original substrate and the segmented cell sheet was estimated with a Python-based implementation that we have used before to quantify curvature distributions of trabecular bone^66^. Briefly, the curvature estimation algorithm locally fits a second order polynomial to a region of the mesh in order to calculate the curvature. The radius of the local sphere used for curvature estimation was set as $r=20\left\langle e\right\rangle$ with $\left\langle e\right\rangle$ the average mesh edge length. This neighborhood size offered an appropriate balance between sufficiently smooth and sufficiently localized curvature estimation. After computing the area-normalized curvatures at every mesh vertex, the meshes were color-coded by curvature and visualized in the open-source software Paraview (version 5.8).

### Quantification of stress fiber orientation

To determine the dominant stress fiber orientation in an ROI, a custom script based on the Fast Fourier Transform (FFT) was implemented, similar to a previously reported method^39^. Details of the implementation are provided in Supplementary Note 2 and Supplementary Fig. 11. Briefly, the approach involved computing the power spectrum of the FFT applied to a grayscale ROI and detecting the orientation of the dominant band of the elevated power values in the spectrum. This orientation was then converted to the dominant orientation in the grayscale image. The orientation analysis was applied to every sub-image in the rasterized images (Fig. 5). For all the structures, the maps of both principal directions were created (similar to the curvature maps described before). The DA between the SF and the first principal direction (pd, corresponding to ${\kappa }_{2}$) was defined as:

3 $${DA}=1-\frac{\angle ({{{{{\bf{sf}}}}}}{{{{{\boldsymbol{,}}}}}}{{{{{\bf{pd}}}}}})}{90}$$

where $\angle ({{{{{\bf{sf}}}}}}{{{{{\boldsymbol{,}}}}}}{{{{{\bf{pd}}}}}})\in ({{{{\mathrm{0,90}}}}})$ indicates the angular difference between the SF and the principal direction.

### Statistics and reproducibility

Unless otherwise indicated in the figure captions, at least three independent experiments were performed for every condition with similar results. Representative confocal micrographs are shown in the figures. All statistical analyses were performed using GraphPad Prism 8.4.2 (GraphPad Software, CA, USA). For all the relevant figures, the type of the data presented, the choice of the statistical tests, and the significance levels are all indicated in the corresponding figure captions.

### Reporting summary

Further information on research design is available in the Nature Portfolio Reporting Summary linked to this article.

## Supplementary information

Supplementary Information

Description of additional Supplementary File

Supplementary Video 1

Supplementary Video 2

Supplementary Video 3

Supplementary Video 4

Supplementary Video 5

Supplementary Video 6

Supplementary Video 7

Supplementary Video 8

Supplementary Video 9

Reporting Summary

## Data Availability

All relevant data supporting the findings of this study are contained within the manuscript and its Supplementary Information file. Raw confocal microscopy z-stacks are readily available from the corresponding author upon reasonable request. Source data are provided with this paper.

## Source data

Source data

## Code availability

The Matlab codes used for processing the microscopy images and analyzing cell patterning with respect to substrate curvature, as well as example microscopy data, are available at DOI: 10.4121/21915963.
