# Supplementary material for: Emergent collective organization of bone cells in complex curvature fields
Source: Nat Commun. 2023 Mar 3;14:855. doi: 10.1038/s41467-023-36436-w (PMC9984480; doi:10.1038/s41467-023-36436-w)
Supplement: Supplementary file 1 — Supplementary Information [file 41467_2023_36436_MOESM1_ESM.pdf]

Supplementary Information to

Emergent collective organization of bone cells in complex curvature fields

Sebastien J.P. Callens[1,2,*], Daniel Fan[3], Ingmar A.J. van Hengel[1], Michelle Minneboo[1], Pedro J. Díaz Payno[1,4], Molly M. Stevens[2], Lidy E. Fratila-Apachitei[1], Amir A. Zadpoor[1]

[1] Department of Biomechanical Engineering, Delft University of Technology (TU Delft), Mekelweg 2, Delft 2628CD, The Netherlands

[2] Department of Materials, Department of Bioengineering, and Institute of Biomedical Engineering, Imperial College London, London SW7 2AZ, United Kingdom

[3] Department of Precision and Microsystems Engineering, Delft University of Technology (TU Delft), Mekelweg 2, Delft 2628CD, The Netherlands

[4] Department of Orthopedics and Sports Medicine, Erasmus MC University Medical Center, Rotterdam 3015GD, The Netherlands

[*] Corresponding author: s.callens21@imperial.ac.uk

Supplementary Note 1: Surface parametrization and curvature

The curvatures of the substrates can be calculated analytically using the expressions provided below. For completeness, we first provide the relationship between the principal curvatures (κ_1 and κ_2) and the mean (H) and Gaussian (K) curvature:

$$H = \frac{1}{2}(\kappa_1 + \kappa_2)$$
$$K = \kappa_1 \kappa_2$$

The inverse relationships are given as:

$$\kappa_1 = H + \sqrt{H^2 - K}$$
$$\kappa_2 = H - \sqrt{H^2 - K}$$

1.1. Cylinder

The constant principal curvatures of the cylindrical substrate are given by:

$$\kappa_1 = \frac{1}{r_{cylinder}}$$
$$\kappa_2 = 0$$

where $r_{cylinder}$ is the radius of the cylinder. In our case, $r_{cylinder} = 90$ µm.

1.2. Unduloid

The unduloid surface is parametrized as [1]:

$$\boldsymbol{x}(u, v) = (x(u), z(u)\cos(v), z(u)\sin(v))$$

with $u \in \mathbb{R}$ and $0 \leq v \leq 2\pi$. In this parametrization, $x(u)$ and $z(u)$ are given by:

$$x(u) = r_1 F\left(\frac{u}{r_1 + r_2} - \frac{\pi}{4}, \frac{r_2^2 - r_1^2}{r_2^2}\right) + r_2 E\left(\frac{u}{r_1 + r_2} - \frac{\pi}{4}, \frac{r_2^2 - r_1^2}{r_2^2}\right)$$
$$z(u) = \sqrt{\frac{r_2^2 - r_1^2}{2} sin\left(\frac{2u}{r_1 + r_2}\right) + \frac{r_2^2 + r_1^2}{2}}$$

where r_1 and r_2 are the smallest and largest radii of the unduloid, respectively. In our experiments, $r_1 = 45$ µm and $r_2 = 135$ µm. In these expressions, F and E respectively represent the incomplete elliptic integrals of the first and second kind, provided by:

$$F(\phi, k) = \int_0^{\phi} \frac{d\theta}{\sqrt{1 - k^2 \sin^2(\theta)}}$$
$$E(\phi, k) = \int_0^{\phi} \sqrt{1 - k^2 \sin^2(\theta)}\, d\theta$$

The unduloid is characterized by a constant mean curvature, which is defined as:

$$H = \frac{1}{r_1 + r_2}$$

The Gaussian curvature of the unduloid varies as a function of $z(u)$:

$$K = \frac{1 - \left(\frac{r_1 r_2}{z(u)^2}\right)^2}{(r_1 + r_2)^2}$$

1.3. Spheres

The principal curvatures on the surface of the spheres are constant and equal:

$$\kappa_1 = \kappa_2 = \frac{1}{r_{spher}}$$

where r_{spher} is the radius of a single sphere. For our experiments, $r = 180$ µm.

1.4. Catenoids

The catenoids in the catenoid-substrate are parametrized by:

$$\boldsymbol{x}(u, v) = (x(u, v), y(u, v), z(v))$$

where $u \in [0,2\pi)$, $v \in \mathbb{R}$, and:

$$x(u, v) = r \cosh\left(\frac{v}{r}\right) \cos(u)$$
$$y = r \cosh\left(\frac{v}{r}\right) \sin(u)$$
$$z = v$$

In these expressions, r is a non-zero, real parameter describing the catenoid neck radius. In our case, $r = 45$ µm and -79.32 µm $< v <$ 79.32 µm. The principal curvatures of the catenoid are given by:

$$k_1 = \frac{1}{r} \operatorname{sech}^2(v)$$
$$\kappa_2 = -\frac{1}{r} \operatorname{sech}^2(v)$$

which, indeed, leads to the defining characteristic of the catenoid as a minimal surface (*i.e.*, $H = 0$).

1.5. Pseudospheres

The pseudospheres (or tractricoids) in the corresponding substrate are parametrized by:

$$\boldsymbol{x}(u, v) = (x(u, v), y(u, v), z(u))$$

where $u \in (-\infty, \infty)$, $v \in [0,2\pi)$, and:

$$x(u, v) = r \operatorname{sech}(u) \cos(v)$$
$$y(u, v) = r \operatorname{sech}(u) \sin(v)$$
$$z(u) = r\,(u - \tanh(u))$$

Here, r defines the mean pseudosphere radius. For our substrates, a section of the infinitely extending pseudosphere was defined by $r = 180$ µm and $u \in [0.795, 2.0635]$. The principal curvatures of the pseudosphere are defined as:

$$\kappa_1 = \frac{\operatorname{sech}(u)}{r \tanh(u)}$$
$$\kappa_2 = -\frac{\tanh(u)}{r \operatorname{sech}(u)}$$

Multiplying these expressions for the principal curvatures, indeed, results in the constant Gaussian curvature of $K = -\frac{1}{r^2}$, which is the defining characteristic of the pseudosphere.

1.6. Sinusoidal cylinder

The sinusoidal cylinder is defined by creating a sinusoidal wave and "extruding" a cylinder along the sinusoidal path. The footprint of the sinusoidal hemi-cylinder is defined by the parallel waves to a standard sine wave. A sine wave can be parametrized as:

$$x(t) = t$$
$$y(t) = a\sin(\omega t)$$

where $t \in \mathbb{R}$, and a and ω are the constants that define the amplitude and frequency of the wave. In our case, $t \in [0,2600]$, $a = 200$, and $\omega = \frac{\pi}{650}$. A parallel sine wave, at a signed distance l ($l \in [-90,90]$) from the original wave, is parametrized by:

$$x(t,l) = t + \frac{l\,a\,\omega\cos(\omega t)}{\sqrt{1 + a^2\omega^2\cos^2(\omega t)}}$$
$$y(t,l) = a\sin(\omega t) - \frac{l}{\sqrt{1 + a^2\omega^2\cos^2(\omega t)}}$$

One of the principal curvatures of the sinusoidal cylinder is equal to the reciprocal of the cylinder radius (in our case $r_{cylinder} = 90$):

$$\kappa_1 = \frac{1}{r_{cylinder}}$$

The other principal curvature is the reciprocal of the radius of curvature of the parallel sine wave at a particular point. For example, for the points on the centreline of the sinusoidal cylinder, the principal curvature k_2 is equal to the reciprocal of the radius of curvature of the original sine wave (with $l = 0$). The radius of curvature for a (parallel) curve $(x(t,l), y(t,l))$ is given by:

$$R(t,l) = \frac{\left(\left(\frac{dx}{dt}\right)^2 + \left(\frac{dy}{dt}\right)^2\right)^{\frac{3}{2}}}{\frac{dx}{dt}\frac{d^2y}{dt^2} - \frac{dy}{dt}\frac{d^2x}{dt^2}}$$

For the specific case of our experiments, the components of this expression are given as:

$$\frac{dx}{dt} = 1 + d\cdot\left(\frac{32\pi^4\sin\left(\frac{\pi t}{650}\right)\cos^2\left(\frac{\pi t}{650}\right)}{714025\left(\frac{16}{169}\pi^2\cos^2\left(\frac{\pi t}{650}\right) + 1\right)^{\frac{3}{2}}} - \frac{2\pi^2\sin\left(\frac{\pi t}{650}\right)}{4225\sqrt{\frac{16}{169}\pi^2\cos^2\left(\frac{\pi t}{650}\right) + 1}}\right)$$

$$\frac{dy}{dt} = \frac{4\pi}{13}\cos\left(\frac{\pi t}{650}\right) - d\cdot\frac{8\pi^3\sin\left(\frac{\pi t}{650}\right)\cos\left(\frac{\pi t}{650}\right)}{25\left(16\pi^2\cos^2\left(\frac{\pi t}{650}\right) + 169\right)^{\frac{3}{2}}}$$

$$\frac{d^2x}{dt^2} = \frac{4\pi d}{13}\left(-\frac{\pi^2 cos\left(\frac{\pi\ t}{650}\right)}{422500\sqrt{\left(\frac{16}{169}\pi^2 cos^2\left(\frac{\pi\ t}{650}\right)\ +\ 1\right)}} - \frac{\left(8\,\pi^4 sin^2\left(\frac{\pi\ t}{650}\right) cos\left(\frac{\pi\ t}{650}\right)\right)}{17850625\ \left(\frac{16}{169}\pi^2 cos^2\left(\frac{\pi\ t}{650}\right) +\ 1\right)^{\frac{3}{2}}}\right.$$

$$\left. +\ cos\left(\frac{\pi\ t}{650}\right)\left(\frac{4\,\pi^4 cos^2\left(\frac{\pi\ t}{650}\right)}{17850625\ \left(\frac{16}{169}\pi^2 cos^2\left(\frac{\pi\ t}{650}\right) +\ 1\right)^{\frac{3}{2}}} + \frac{192\,\pi^6 sin^2\left(\frac{\pi\ t}{650}\right) cos^2\left(\frac{\pi\ t}{650}\right)}{3016755625\ \left(\frac{16}{169}\pi^2 cos^2\left(\frac{\pi\ t}{650}\right) +\ 1\right)^{\frac{5}{2}}} - \frac{4\,\pi^4 sin^2\left(\frac{\pi\ t}{650}\right)}{17850625\ \left(\frac{16}{169}\pi^2 cos^2\left(\frac{\pi\ t}{650}\right) +\ 1\right)^{\frac{3}{2}}}\right)\right)$$

$$\frac{d^2y}{dt^2} = \frac{-2\pi^2}{4225\sin\left(\frac{\pi t}{650}\right)} - d\left(\left(\frac{4\,\pi^4 cos^2\left(\frac{\pi\ t}{650}\right)}{17850625\ \left(\frac{16}{169}\pi^2 cos^2\left(\frac{\pi\ t}{650}\right) +\ 1\right)^{\frac{3}{2}}} + \frac{192\,\pi^6 sin^2\left(\frac{\pi\ t}{650}\right) cos^2\left(\frac{\pi\ t}{650}\right)}{3016755625\ \left(\frac{16}{169}\pi^2 cos^2\left(\frac{\pi\ t}{650}\right) +\ 1\right)^{\frac{5}{2}}} - \frac{4\,\pi^4 sin^2\left(\frac{\pi\ t}{650}\right)}{17850625\ \left(\frac{16}{169}\pi^2 cos^2\left(\frac{\pi\ t}{650}\right) +\ 1\right)^{\frac{3}{2}}}\right)\right)$$

Using these components, the radius of curvature $R(t, l)$ can be determined, which is then used to calculate the second principal curvature on the surface of the sinusoidal cylinder:

$$\kappa_2 = \frac{1}{R(t, l)}$$

Supplementary Note 2: FFT-based calculation of principal image orientation

The principal orientations in the stack projections were determined using an image processing approach based on the discrete Fourier transform (DFT). First, the image, or image section in case of a domain-wise analysis, was multiplied by a cosine-shaped windowing function (Supplementary Figure 11a-b) to avoid artefacts in the power spectrum of the DFT that would otherwise appear due to the image boundaries (the DFT assumes periodicity at the image boundaries, which is not the case in general). Next, the fast Fourier transform (FFT) was applied and the power spectrum P was calculated as:

$$P = \log(|Y| + 1)$$

where Y is the complex result of the FFT, shifted such that the low frequencies are situated in the centre of the spectrum (Supplementary Figure 5c). The oriented components in the original image appear as oriented lines or ellipses in the power spectrum (albeit rotated by $\pi/2$). The power spectrum was rotated in the range $[0, \pi]$ and the sum of the power in the central columns was calculated for every instance of the rotated spectrum ($\sum P_c(\alpha)$). This operation resulted in a $\sum P_c(\alpha)$ *vs,* α curve, in which several peaks could be detected (Supplementary Figure 5d). The principal orientation in the power spectrum was found at the peak with the maximum prominence. To determine the strength of the orientation, the maximum prominence of the curve was divided by the sum of the prominences of all the peaks in the $\sum P_c(\alpha)$ *vs.* α curve. To visualize the orientation distribution in the domain-wise analyses, the vectors were scaled by the orientation strength and mean intensity for that domain.

SUPPLEMENTARY FIGURE 1

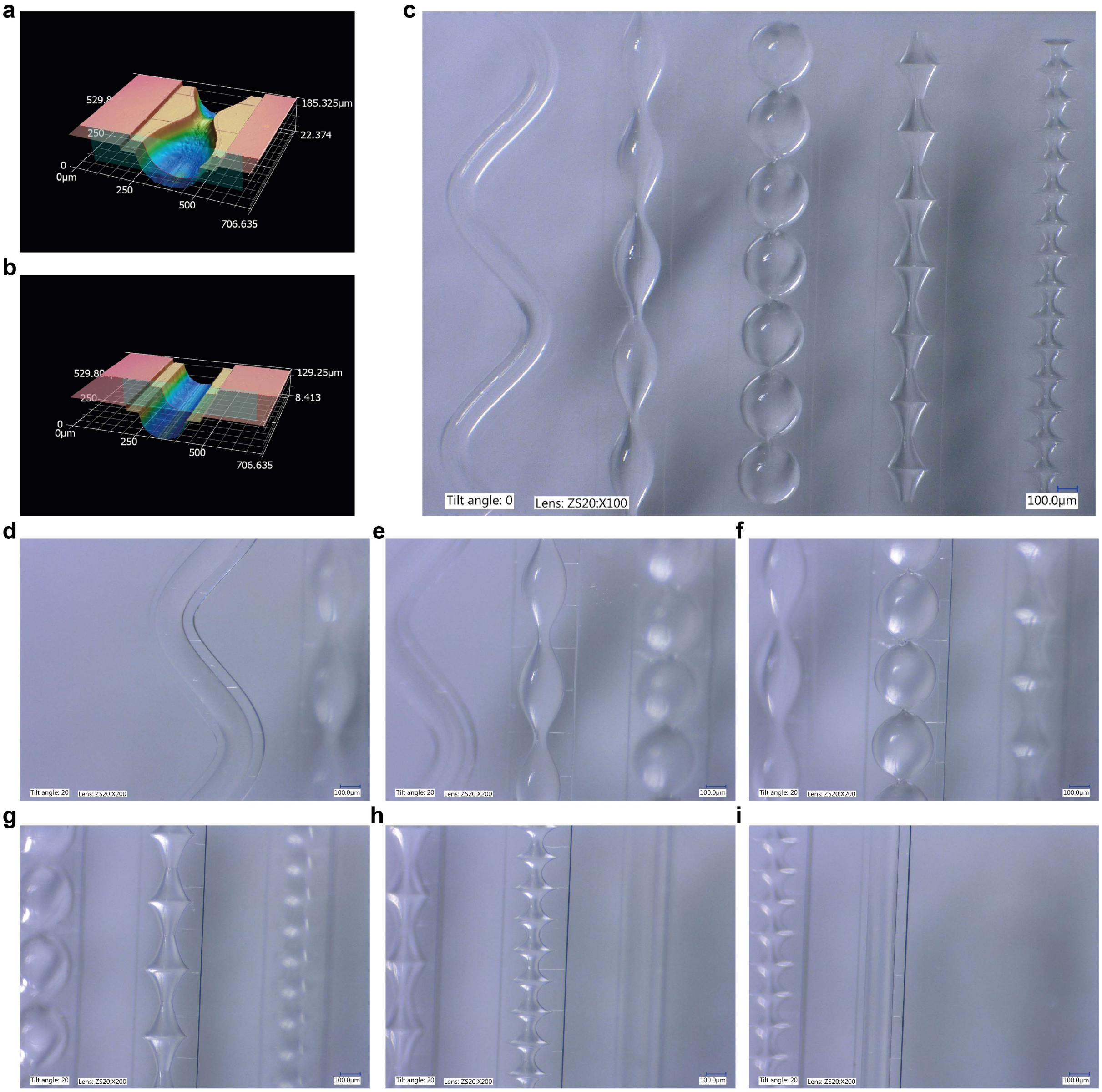

Supplementary Figure 1: Examples of PDMS cell culture substrates. a-b) Representative 3D reconstructions of the concave unduloid (a) and cylinder (b) PDMS substrates, obtained using laser profilometry. c) Representative light microscopy image of convex PDMS substrates obtained using a Keyence VHX-6000 digital microscope. d-i) Higher magnification light microscopy images of the convex PDMS substrate. d) Sinusoidal cylinder, e) unduloid, f) spheres, g) pseudospheres, h) catenoids, i) cylinder.

SUPPLEMENTARY FIGURE 2

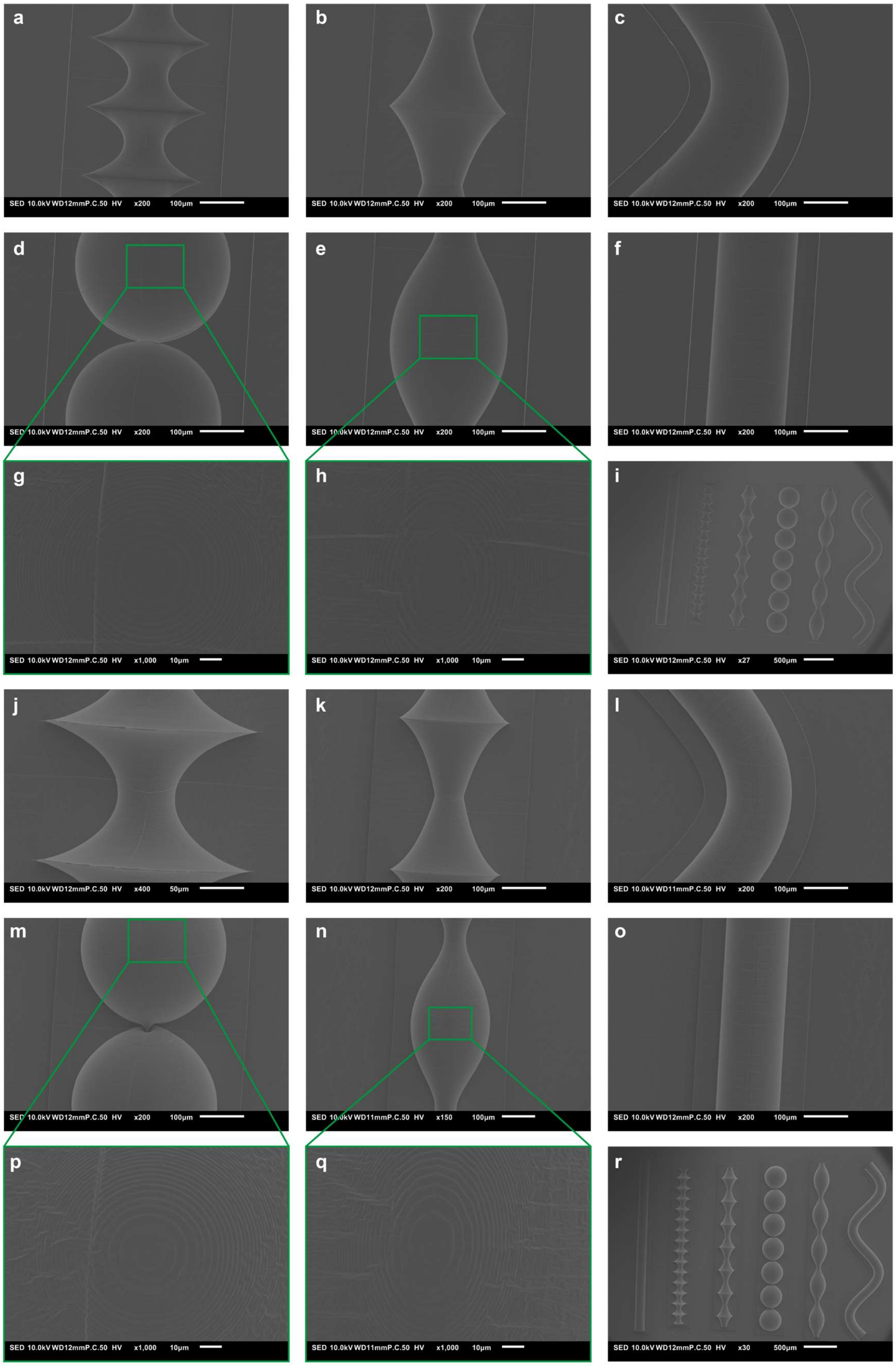

Supplementary Figure 2: Scanning electron microscopy (SEM). a-i) Representative SEM images of the concave PDMS substrates at different magnifications (indicated in every image). j-r) SEM images of the convex PDMS substrates.

Supplementary Figure 3

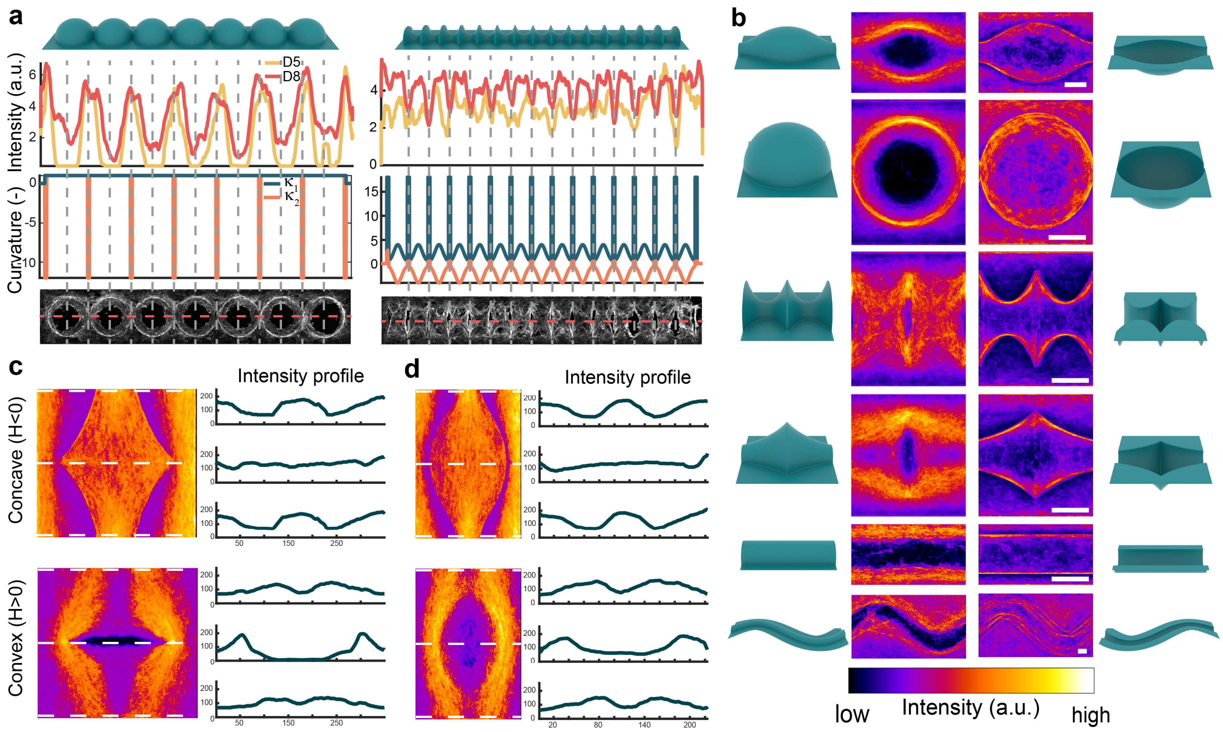

Supplementary Figure 3: Spatial cell patterning on curved substrates. a) Normalized F-actin intensity at the midline of the convex spherical (left) and convex catenoidal (right) substrates. The normalized principal curvatures are indicated in the centre row plots. b) Normalized F-actin frequency maps for the convex (left) and concave (right) substrates on day 5. The scale bars represent 100 µm. c) Actin intensity profiles at different locations obtained from the frequency map of the pseudospherical substrates at day 8 for the concave (top) and convex (bottom) variants. d) Similar representation as in c) but for the concave (top) and convex (bottom) unduloid substrates. Data obtained from 3 independent experiments.

Supplementary Figure 4

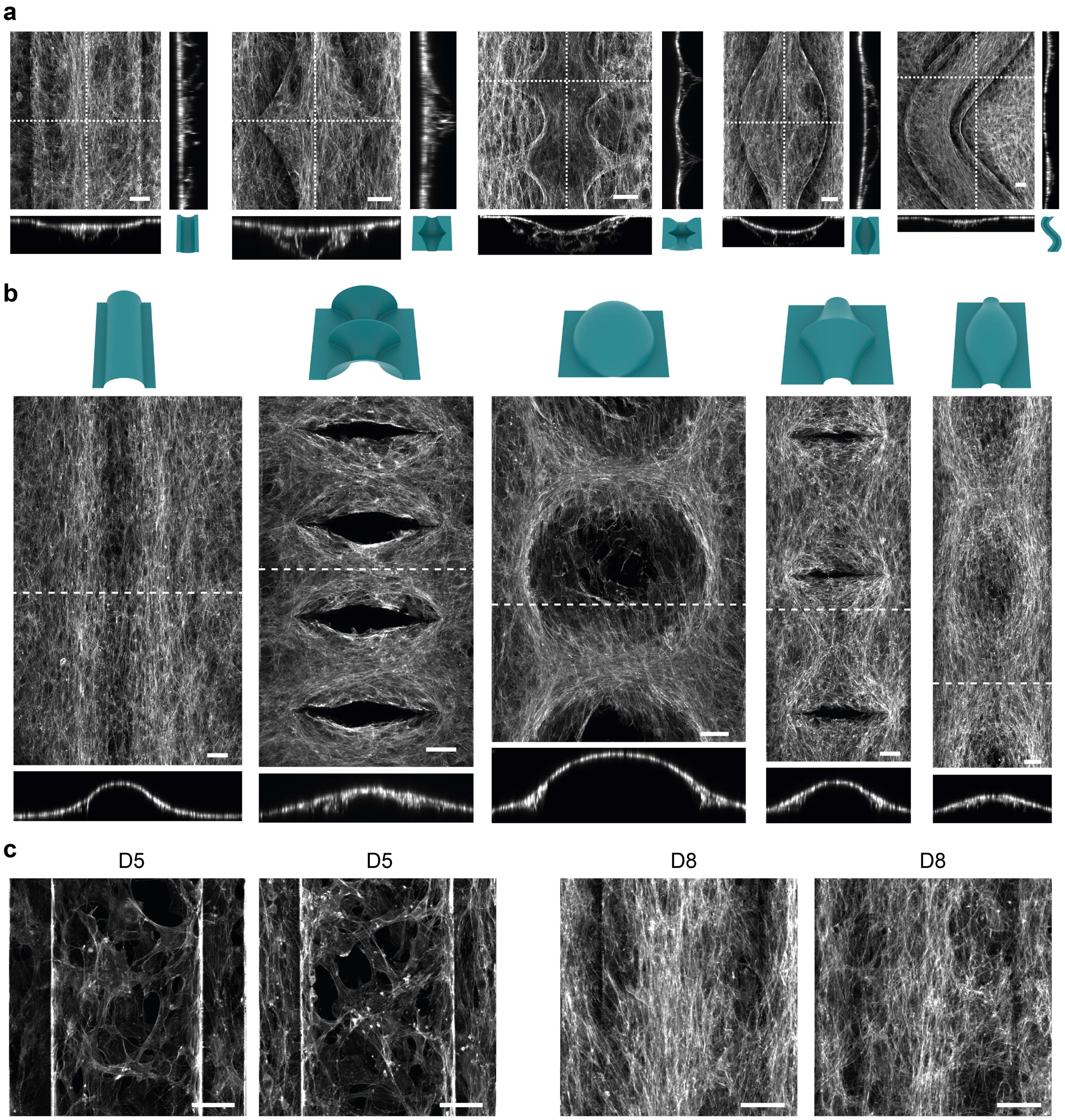

Supplementary Figure 4: Cell sheet bridging on the convex and concave substrates. a) Representative maximum intensity projections and cross-sectional views, showing the formation of cell sheets on the other concave substrates. From left to right: cylinder, pseudospheres, catenoids, unduloid, and sinusoidal cylinder. b) The convex-to-planar transition is a local concavity, which is collectively bridged by the cells. Images depict the maximum intensity projections and orthogonal views of the actin channel for the representative cases on day 8. From left to right: cylinder, catenoids, spheres, pseudospheres, and unduloid. c) Cell bridging on the concave cylindrical substrates. On day 5, the bridges are randomly oriented. On day 8, a longitudinal, collective SF alignment is observed. All scale bars are 50 µm.

SUPPLEMENTARY FIGURE 5

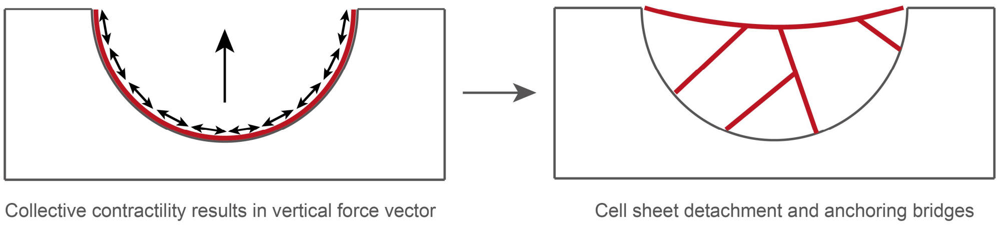

Supplementary Figure 5: Cell sheet formation schematic. Cross-sectional schematic view of a concave channel with cells. When cells initially cover the channel (left, red line), they exert tensile forces on each other due to cell contractility. This results in an upwards pointing resultant force vector that causes the cells to detach from the substrate and mature into a detached cell sheet with anchoring bridges (right).

Supplementary Figure 6

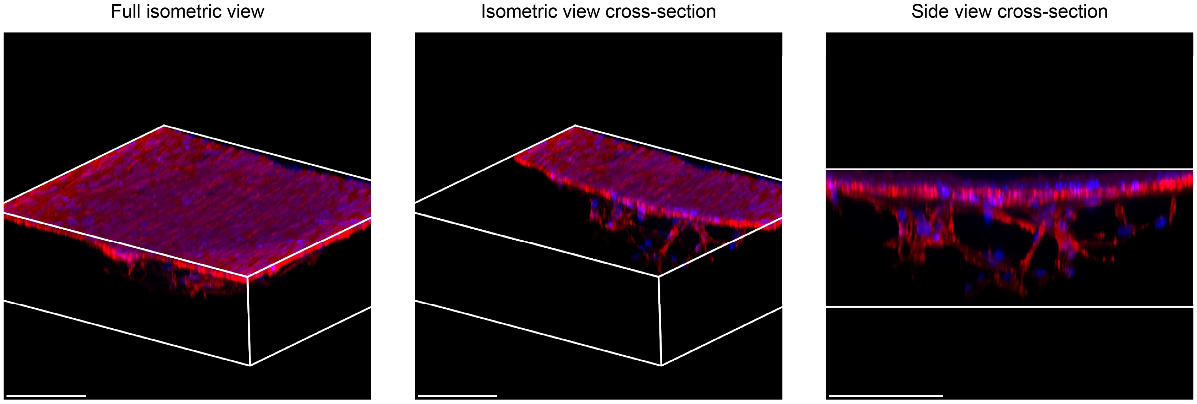

Supplementary Figure 6: Cell sheet and anchor formation. 3D reconstruction of the formation of a detached cell sheet and anchoring cell bridges underneath the sheet over a concave spherical well (Day 8, control medium with TGF-β, stained for F-actin (red) and DNA (blue)). Supplementary Movie 5 provides an animation of this 3D reconstruction. Scale bars represent 100 μm.

Supplementary Figure 7

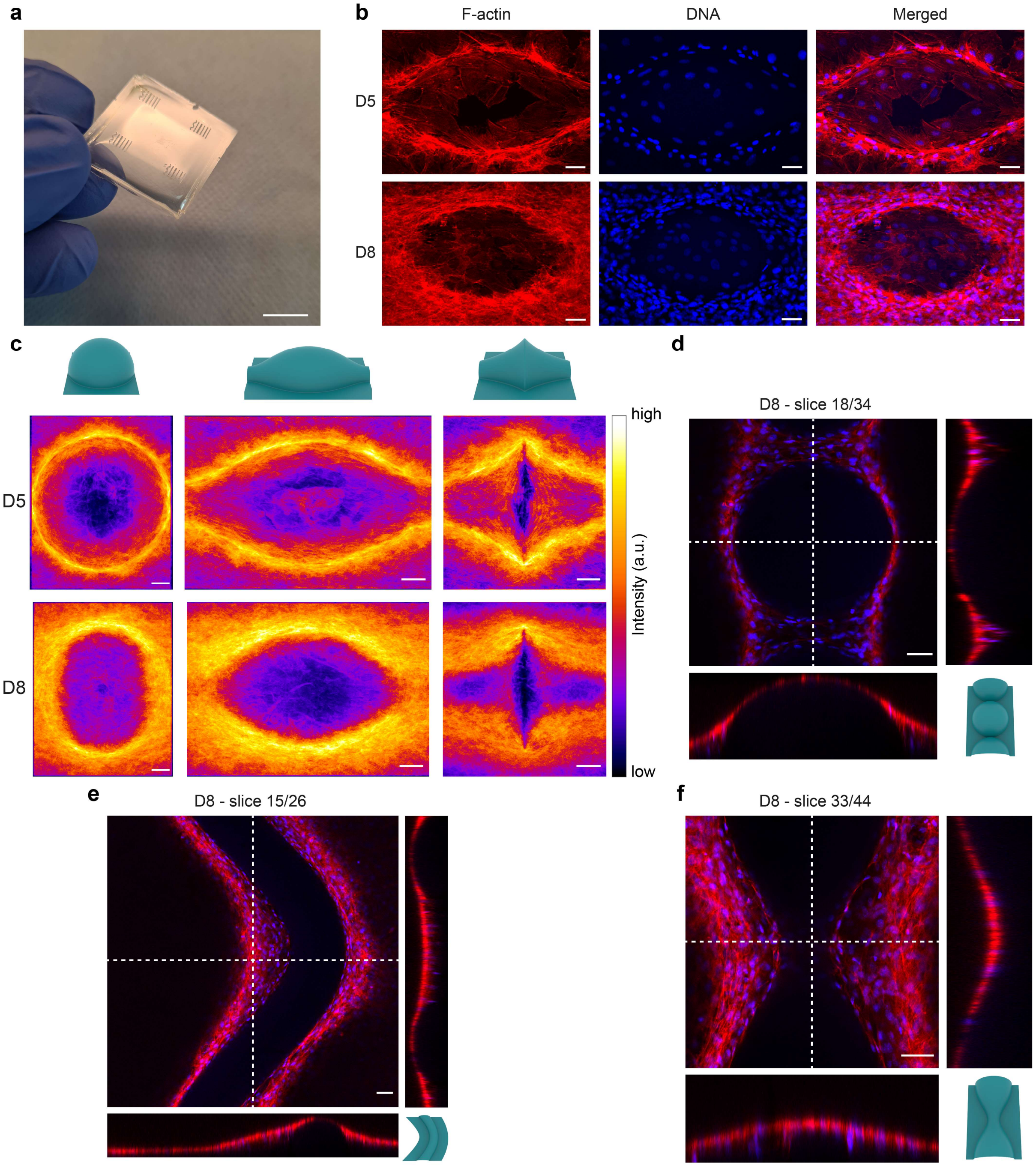

Supplementary Figure 7: Cell organization on convex PS substrates. a) Replica-molded PS substrates of the convex designs. b) Representative maximum intensity projections showing F-actin (red) and DNA (blue) staining for the convex PS unduloid at days 5 and 8. c) Frequency maps displaying spatial F-actin patterning on days 5 and 8 for the convex PS spheres, unduloid, and pseudospheres. The data is obtained by stacking period units from at least two experiments. d-f) Individual z-stack slices and cross-sectional views of F-actin (red) and DNA (blue), showing cell bridging at the convex-to-planar transitions on the sphere (d), sinusoidal cylinder (e), and unduloid substrates (f). The scale bar in a) represents 1 cm. All other scale bars represent 50 µm.

SUPPLEMENTARY FIGURE 8

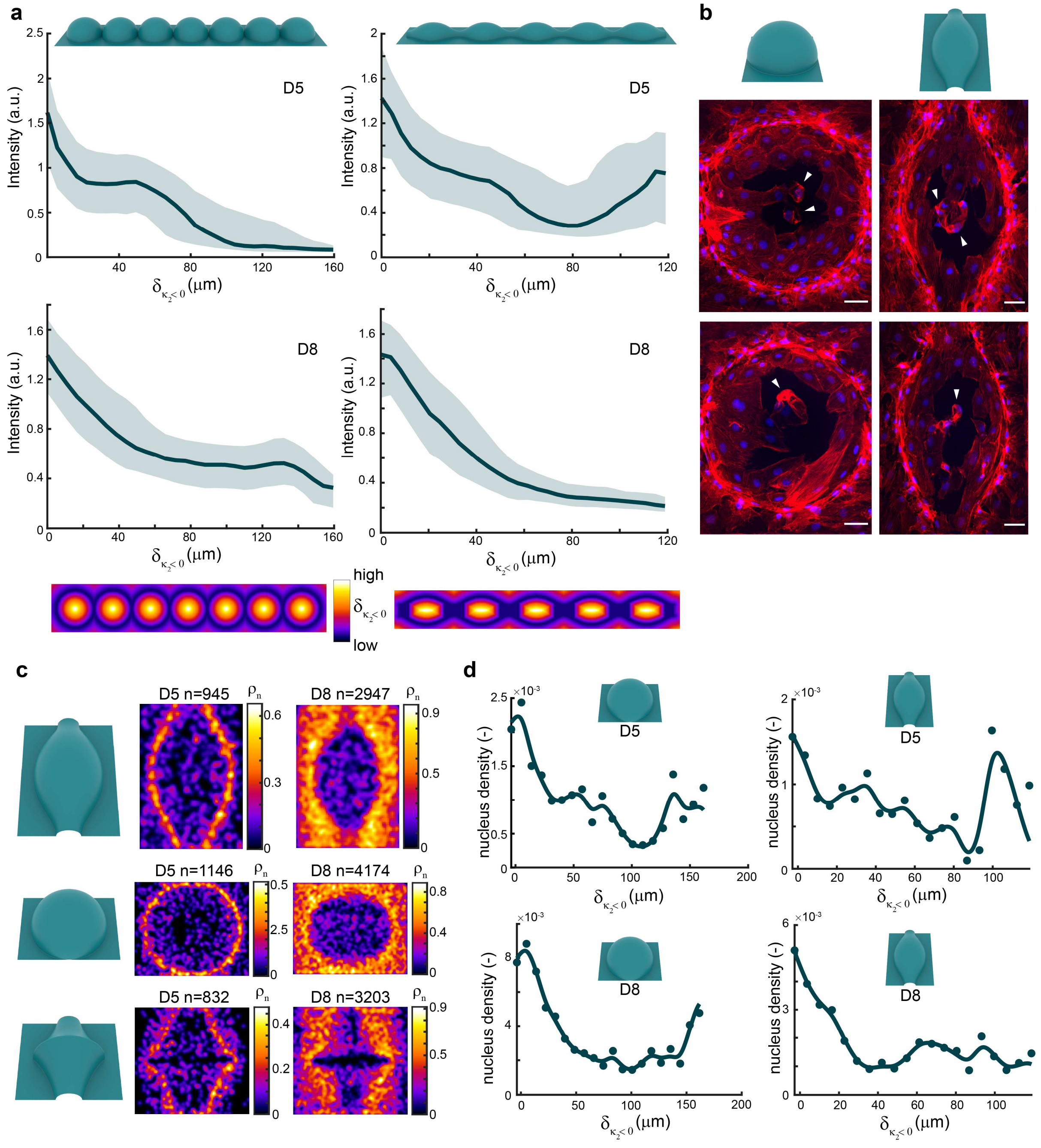

Supplementary Figure 8: Cell patterning as a function of $\boldsymbol{\delta_{\kappa_2<0}}$ on convex PS substrates. a) Normalized F-actin intensity reducing with increasing $\delta_{\kappa_2<0}$, for the convex spheres and unduloid substrates at days 5 and 8. The data is obtained from at least two experimental specimens. The solid line represents the median value and the shaded areas correspond to the interquartile range. The bottom row depicts Euclidean distance maps. b) Maximum intensity projections of F-actin (red) and DNA (blue) on two convex spheres and unduloid substrates at day 5, indicating partial cell-substrate delamination at the regions with positive Gaussian curvature in some cases (white arrows). Scale bars represent 50 µm. c) Frequency maps of nuclei centroids at days 5 and 8 on the convex unduloid, spheres, and pseudospheres (see Figure 2 in the main text). d) Nucleus density *vs.* $\delta_{\kappa_2<0}$ for the convex spheres and unduloid substrates at days 5 and 8 (See Figure 2 in the main text).

SUPPLEMENTARY FIGURE 9

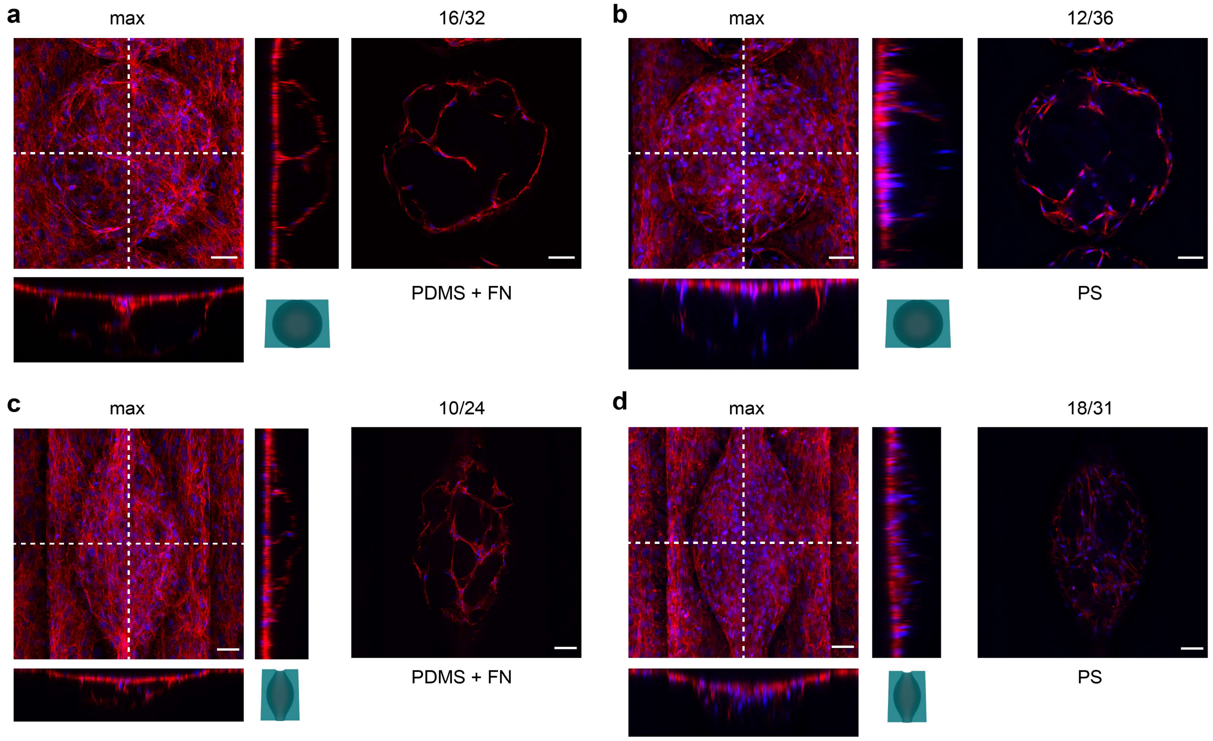

Supplementary Figure 9: Cell bridging on concave PDMS and PS substrates. a-b) Fluorescent staining of F-actin (red) and DNA (blue) on a concave spherical PDMS substrate with adsorbed fibronectin (a) and a concave spherical PS substrate (b) after 8 days in control medium. Left panel is a maximum intensity projection with cross-sectional views. Right panel is a single image throughout the z-stack. c-d) Same as a-b), but for the concave PDMS unduloid with adsorbed fibronectin (c) and the concave PS unduloid (d). All scale bars are 50 µm.

Supplementary Figure 10

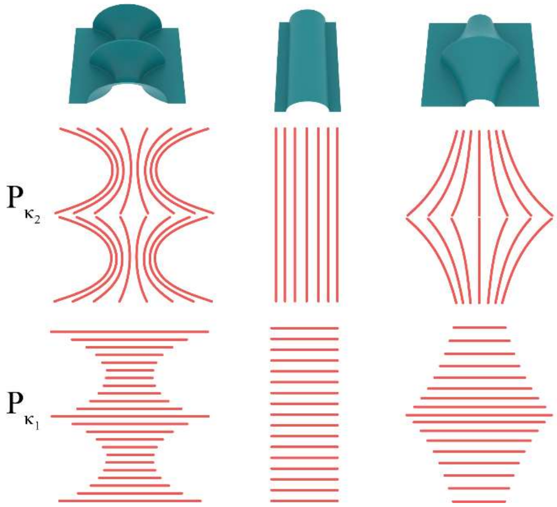

Supplementary Figure 10: Principal directions for curved substrates. The two principal directions P_{κ_2} and P_{κ_1} corresponding to the minimum (κ_2) and maximum (κ_1) principal curvatures, respectively. From left to right: catenoids, cylinder, and pseudospheres.

SUPPLEMENTARY FIGURE 11

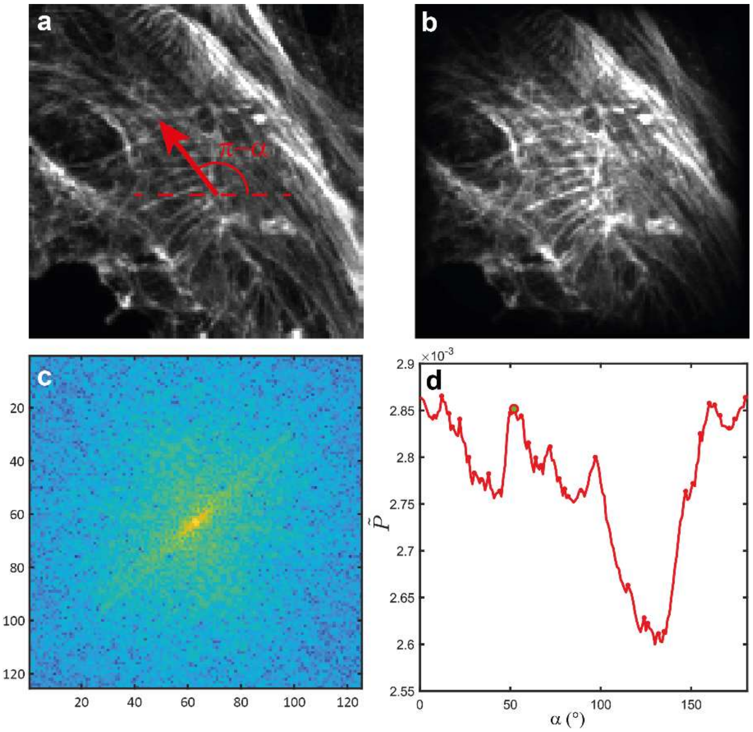

Supplementary Figure 11: FFT-based approach to quantify image orientation. a) Original image section with principal orientation highlighted by the red arrow. b) Image after windowing operation. c) Power spectrum obtained after FFT on the image shown in b). d) $\sum P_c(\alpha)$ *vs.* α curve with peaks highlighted by red markers. The green marker corresponds to the peak with maximum prominence. The corresponding angle α determines the principal orientation in the power spectrum.

SUPPLEMENTARY FIGURE 12

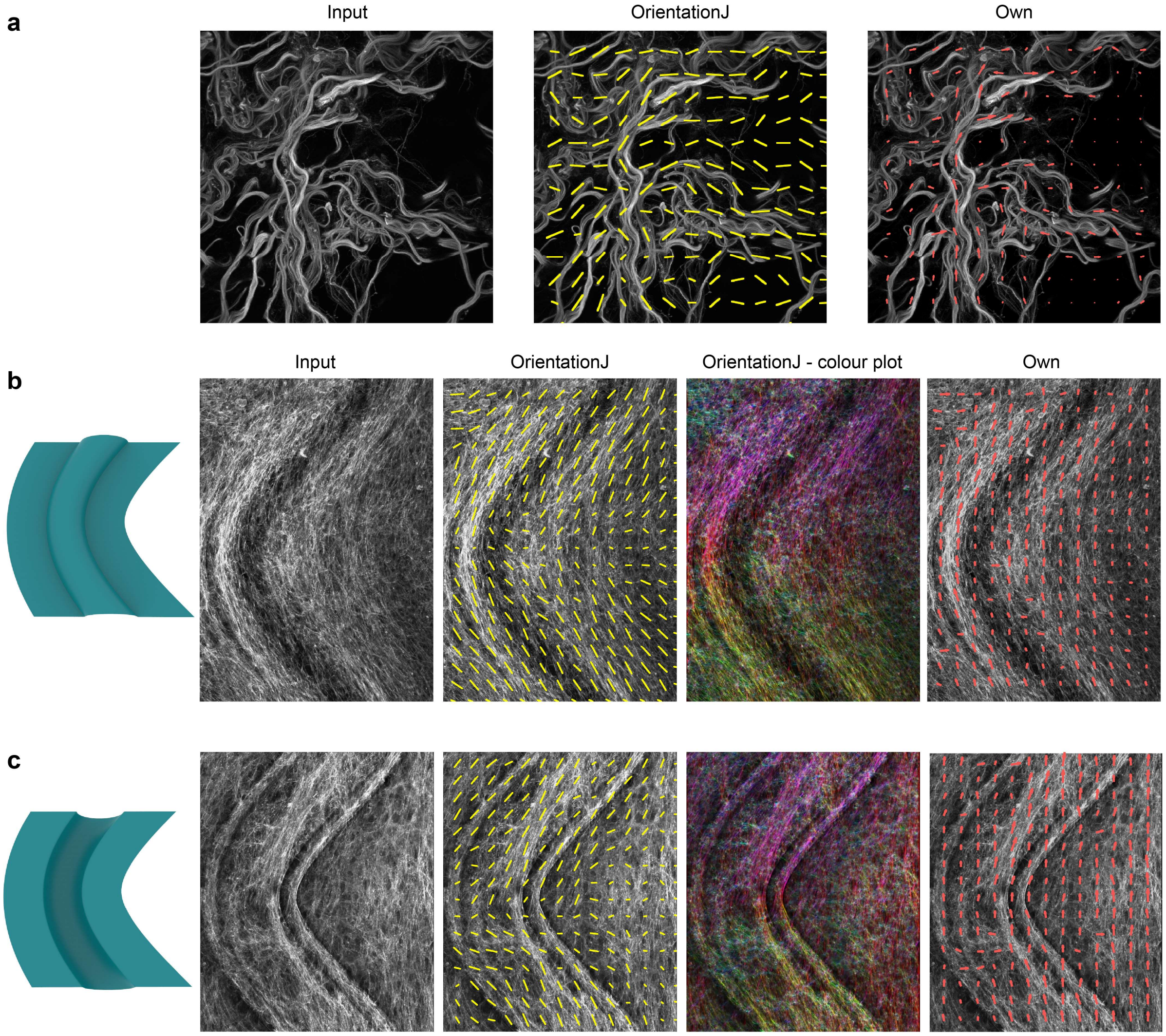

Supplementary Figure 12: Comparison between OrientationJ and own FFT-based approach. a) Comparison between the Fiji-based plugin OrientationJ and the custom FFT-based Matlab tool used in this research, applied to standard benchmarking image (available at http://bigwww.epfl.ch/demo/orientation/). b-c) Comparison between OrientationJ and the custom tool, applied to F-actin maximum intensity projections (day 8) of the convex and concave sinusoidal cylinder, respectively. Both tools show similar results in terms of overall orientation detection, although our custom tool detects the dominant orientation in a grid cell, rather than the average orientation.

SUPPLEMENTARY FIGURE 13

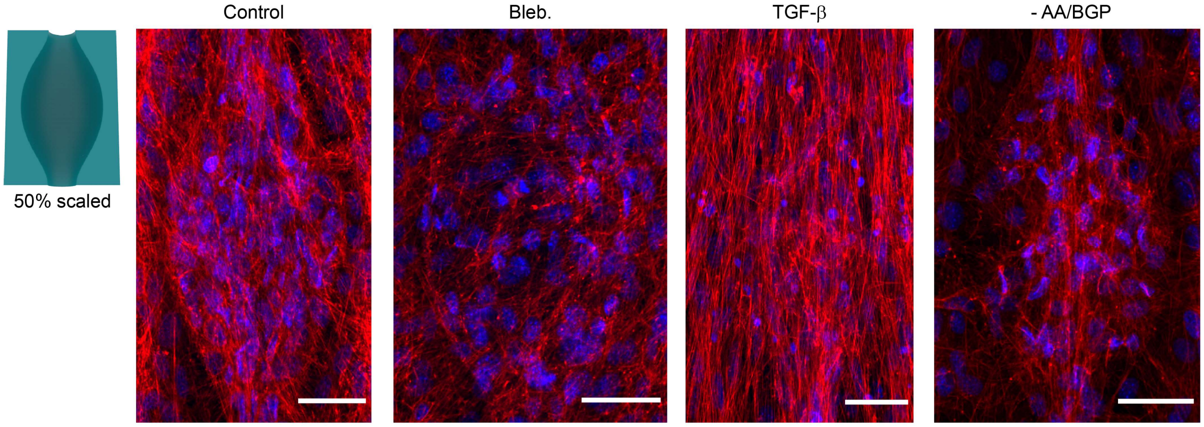

Supplementary Figure 13. Representative maximum intensity projections of F-actin (red) and DNA (blue) at day 8 on the scaled-down concave unduloid (50%), showing the effect of SF morphology and collective orientation under different culture conditions. Scale bars are 50 µm.

SUPPLEMENTARY FIGURE 14

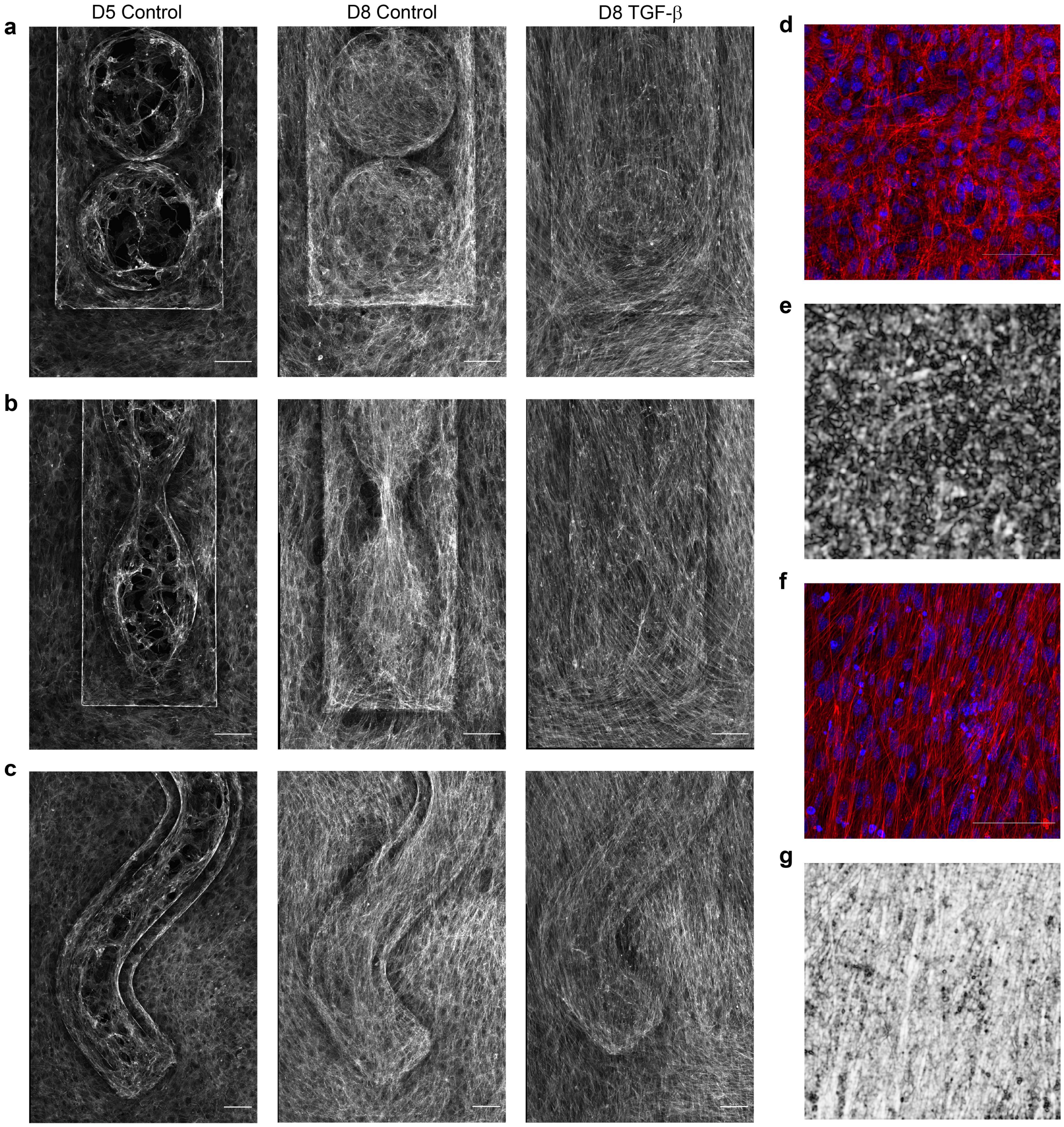

Supplementary Figure 14: Stress fiber orientations outside areas of interest. a) Representative maximum intensity projections of the F-actin channel on the concave spherical substrate at day 5 in control medium (left), day 8 in control medium (middle) and day 8 in control medium supplemented with TGF-β (right), showing the stress fiber organization outside of the primary region of interest (*i.e.* the curved substrates). At day 5, high-intensity and highly aligned stress fibers are visible at the edges of the rectangular domain surrounding the curved substrates. b-c) Same as for a), but for the concave unduloid and concave sinusoidal cylinder substrate, respectively. d) Maximum intensity projection of F-actin (red) and DNA (blue) at day 8 on a flat substrate in control medium. e) Orientation coherence of the image in d), as obtained by OrientationJ. White and black colors indicate high and low coherence, respectively. f-g) Same as d-e), but now for cells at day 8 on a flat substrate in control medium supplemented with TGF-β.

Supplementary Figure 15

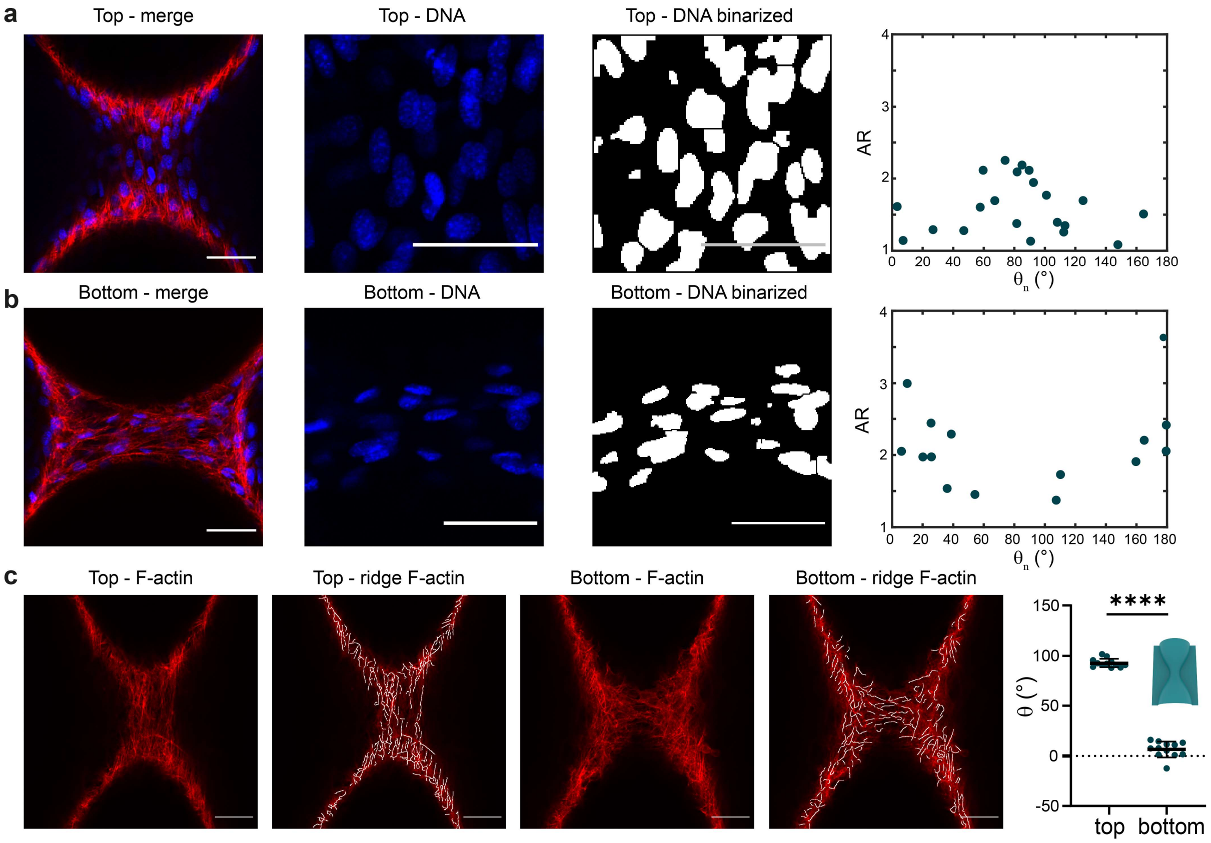

Supplementary Figure 15: Stress fiber subpopulations on saddle-shaped regions of the convex spheres and convex unduloid. a) Single slice from the top of the z-stack on the transition between two convex hemispheres, showing the vertically oriented stress fibres and vertical alignment of the nuclei. The graph on the right indicates the aspect ratio (AR) of the binarized nuclei (separated using a watershed algorithm) versus their orientation θ_n, with 90° corresponding to vertical alignment. b) Same as in a), but no for a slice in the bottom region of the z-stack. c) F-actin orientation and ridge detection (obtained using Fiji) on the top and bottom slices of the neck region of a convex unduloid substrate. The graph on the right displays the average stress fiber orientation in the neck region. n = 12 different examined regions over 3 independent experiments. Data are presented as mean values +/- SD. Paired two-tailed t-tests: **** $p < 0.0001$. Scale bars represent 50 µm.

Supplementary Figure 16

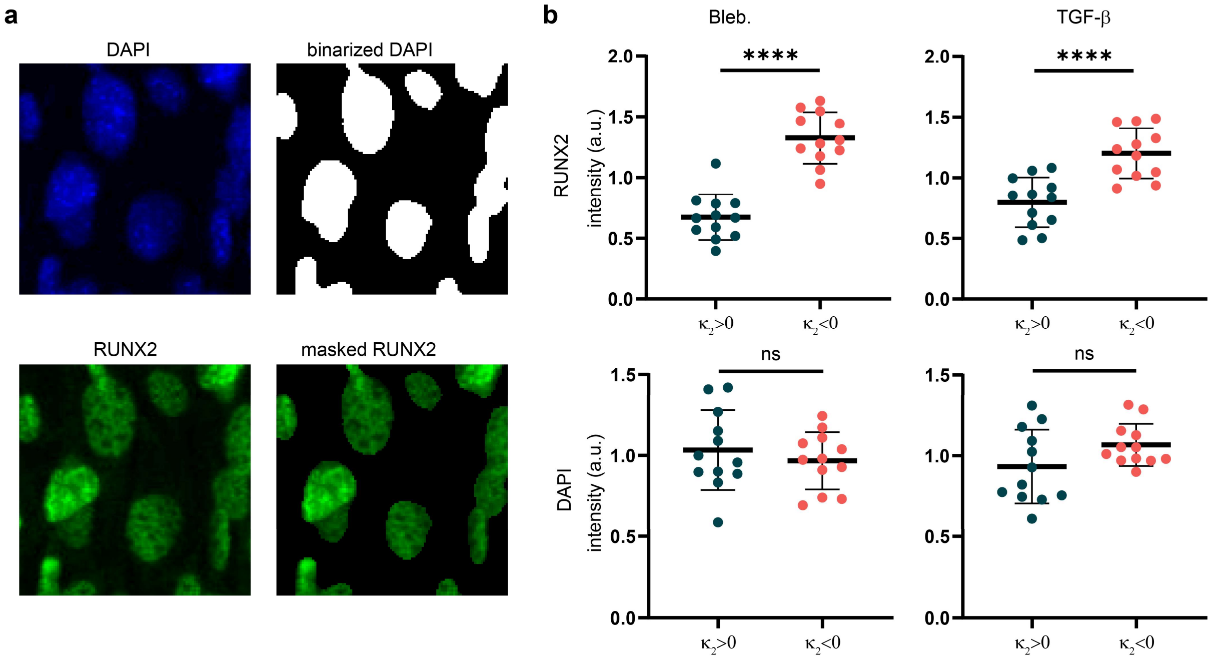

Supplementary Figure 16: RUNX2 quantification and results for cells treated with blebbistatin and TGF-β. a) Workflow to quantify DAPI and RUNX2 intensity. The ROI of the DAPI channel was binarized and used as a mask. Then, the mean intensity of the masked RUNX2 and DAPI images was calculated. b) Similar results as in Figure 6c-d, but now for cells treated with blebbistatin and TGF-β. n = 12 different ROIs from 3 independent experiments. Data are presented as mean values +/- SD. Unpaired two-tailed t-test, ****: $p < 0.0001$, ns: not significant.

Supplementary Figure 17

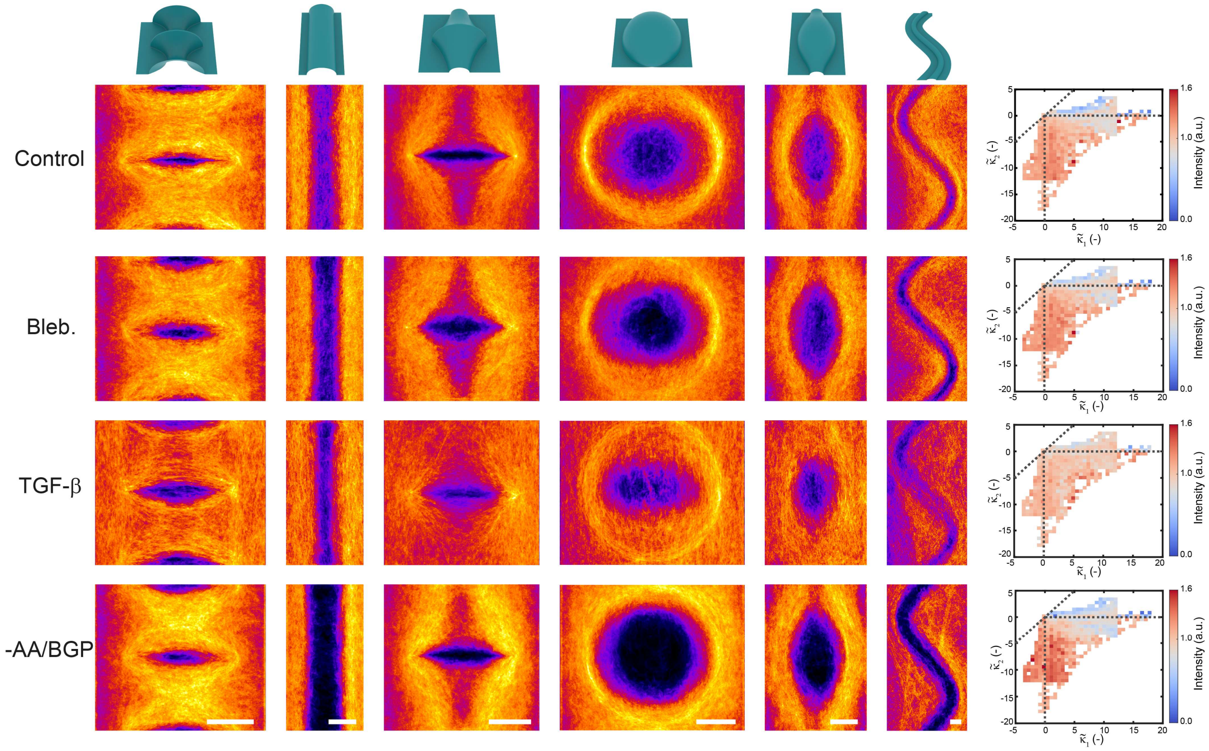

Supplementary Figure 17: Frequency maps and intensity versus curvature. Frequency maps depicting the normalized F-actin signal for all the six convex substrates on day 8. Comparison between the control cells, cells treated with blebbistatin, cells treated with TGF-β, and cells deprived of ascorbic acid (AA) and β-glycerophosphate (GB). The right column shows the heatmaps of the intensity *vs.* the two principal curvatures.

SUPPLEMENTARY FIGURE 18

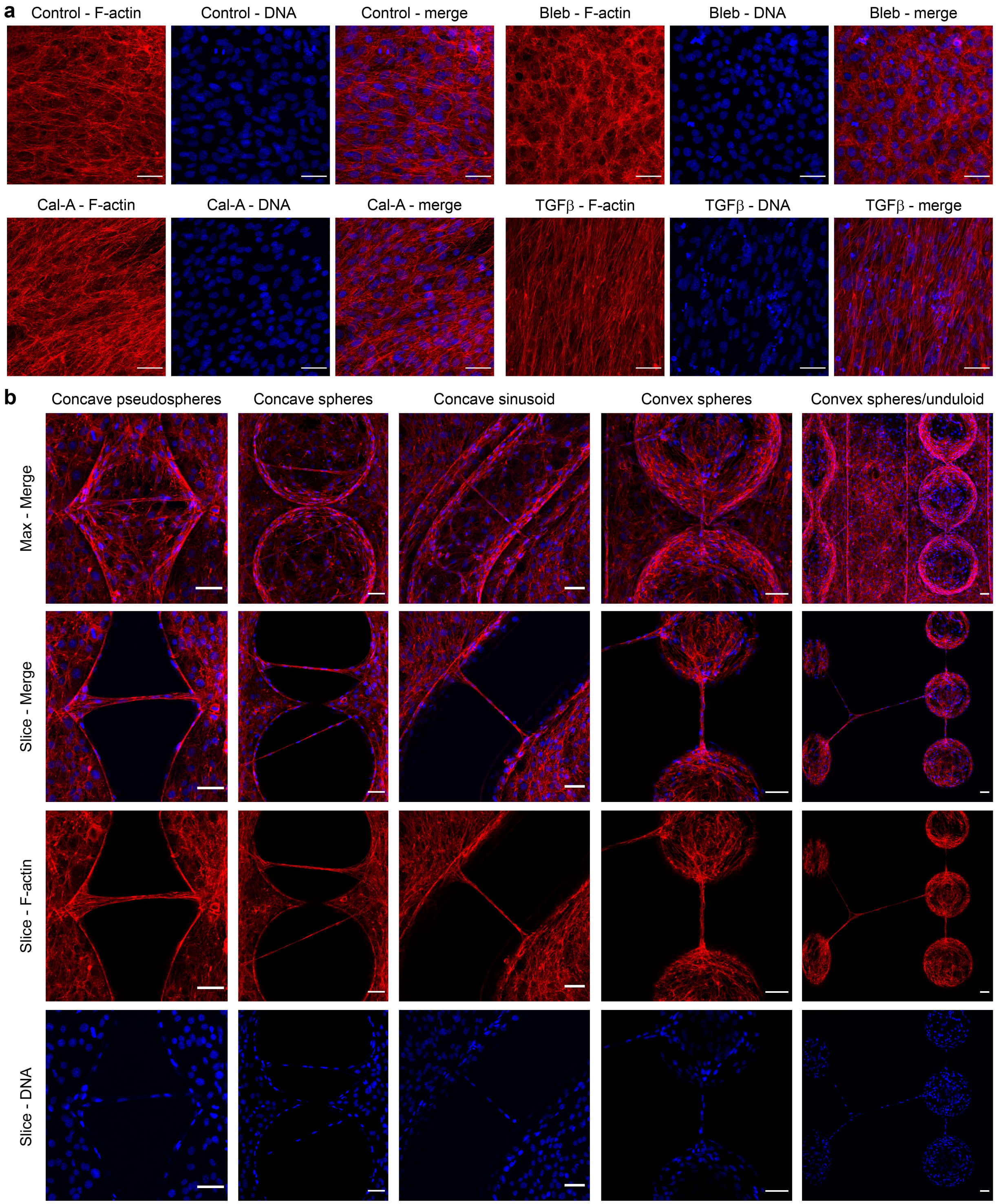

Supplementary Figure 18: Effect of Calyculin-A on stress fiber organization. a) Comparison of cells at day 8 on flat substrates between different culture conditions, showing F-actin, DNA and merged maximum intensity projections. b) Representative maximum intensity projections and slices through the z-stack of large, multicellular cell cables spanning over large concave regions. All scale bars are 50 μm.

SUPPLEMENTARY FIGURE 19

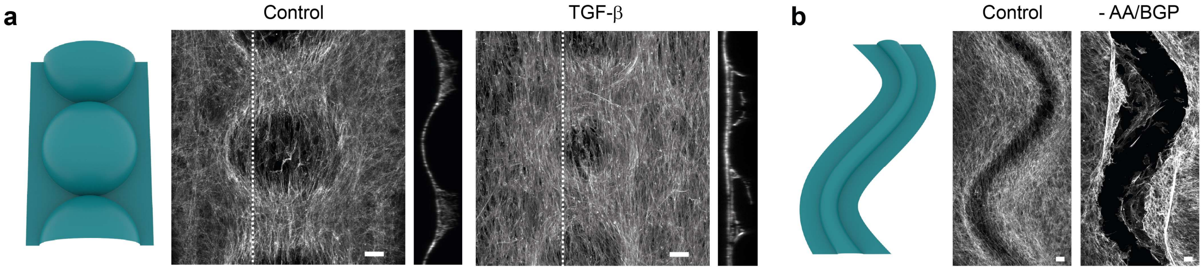

Supplementary Figure 19: Cell sheet formation on convex structures in perturbed conditions. Contractility enhancement affects bridge formation on the convex spherical substrates. A stronger cell sheet is observed in the specimens treated with TGF-β. b) Differentiation inhibition causes cell sheet detachment on the convex sinusoidal cylinders in some specimens. The cells collectively pull away from the concave bend of the substrate. All scale bars are 50 μm.

Supplementary Figure 20

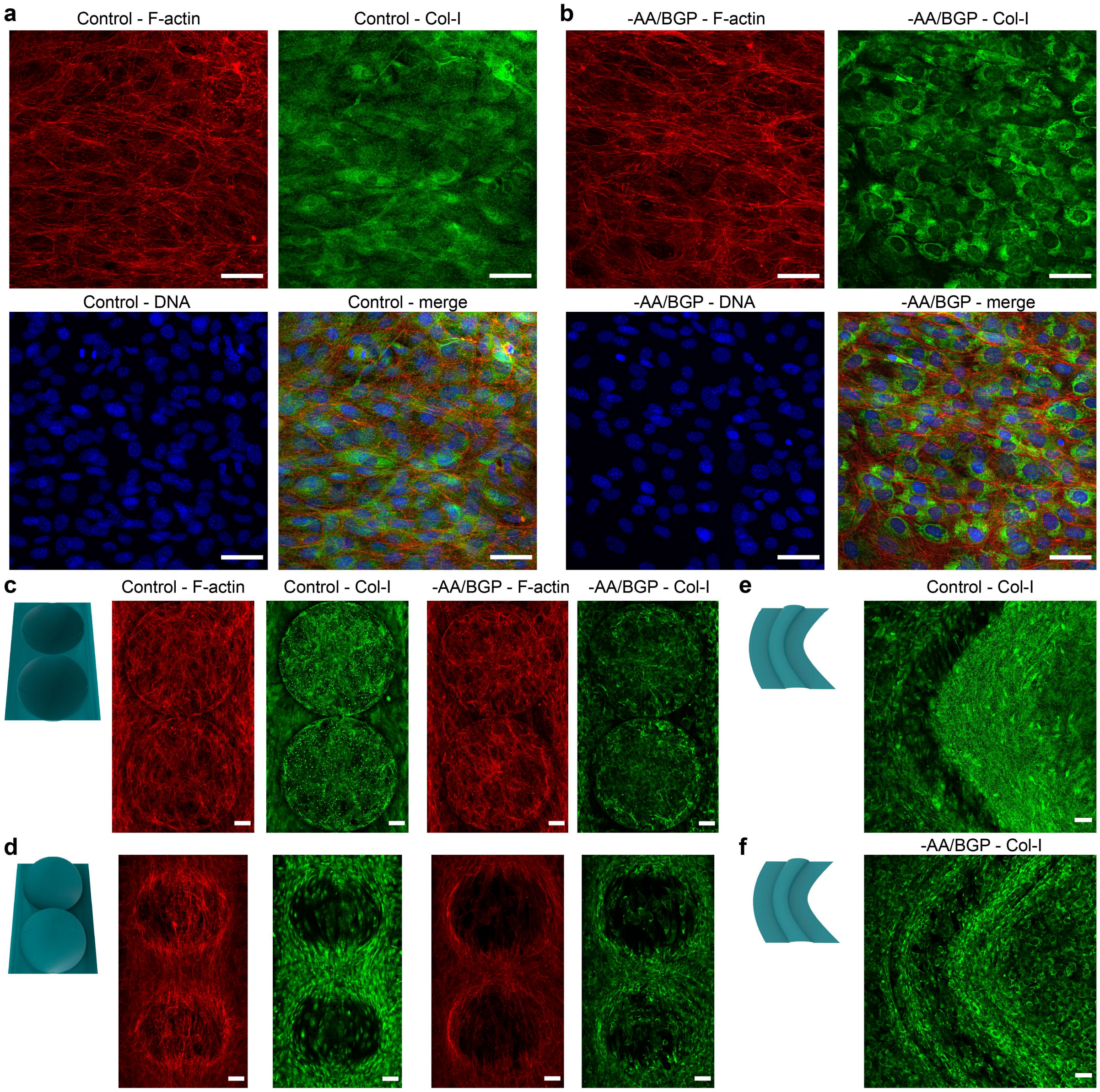

Supplementary Figure 20: Perturbation on convex substrates and col-I expression. a) Expression of F-actin, Col-I and DNA in a control sample on a flat substrate. b) Same as a), but for culture medium without AA and BGP. In this case Col-I is primarily expressed inside the individual cells, surrounding the nucleus. c-d) For concave and convex spherical substrates, respectively: F-actin and col-I expression in control and -AA/BGP culture conditions. e-f) Col-I expression at the bend of a convex sinusoidal wavy substrate in control (top) and -AA/BGP (bottom) conditions. All scale bars are 50 μm.

SUPPLEMENTARY FIGURE 21

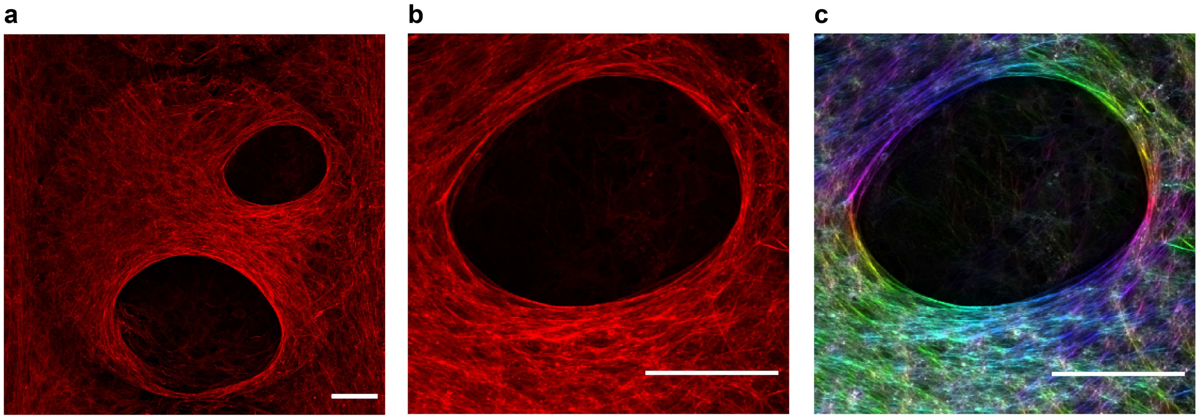

Supplementary Figure 21: Circumferential F-actin alignment at cell sheet hole. a) F-actin maximum intensity projection of cells at day 8 on the 200% scaled concave spherical substrate (image also shown in Figure 4i of the main text). b) Higher magnification view of the smaller hole (top right) in panel a), showing circumferential F-actin organization around the hole. c) The circumferential F-actin alignment in panel b) is confirmed by the colour survey analysis of OrientationJ. All scale bars are 100 µm.

SUPPLEMENTARY FIGURE 22

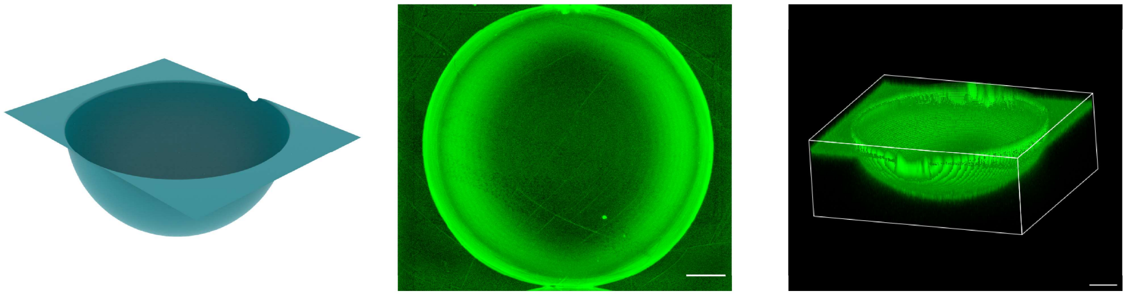

Supplementary Figure 22: Fluorescently labelled FN on concave spherical well. Middle panel shows maximum intensity (z) projection, right panel shows 3D (maximum intensity) reconstruction. All scale bars are 50 µm.

REFERENCES

1. Hadzhilazova, M., *et al.*, *Archivum mathematicum* (2007) **43** (5), 417
