# Supplementary material for: Emergent collective organization of bone cells in complex curvature fields
Source: Nat Commun. 2023 Mar 3;14:855. doi: 10.1038/s41467-023-36436-w (PMC9984480; doi:10.1038/s41467-023-36436-w)
Supplement: Supplementary file 2 — Description of additional Supplementary File [file 41467_2023_36436_MOESM2_ESM.pdf]

Descriptions of additional supplementary files

Supplementary Movie 1. Transformation of the unduloid surface family, interpolating between a cylinder and a set of spheres. The surfaces are coloured by the Gaussian and mean curvatures.

Supplementary Movie 2. 3D reconstruction of the F-actin staining on a concave spherical substrate on day 8, showing cell sheet detachment and anchoring bridges. Scale bar is 100 µm.

Supplementary Movie 3. 3D reconstruction of the F-actin staining on a concave spherical substrate on day 8, for cells treated with blebbistatin. Scale bar is 100 µm.

Supplementary Movie 4. 3D reconstruction of the F-actin staining on a concave spherical substrate on day 8, for cells treated with TGF-β. Scale bar is 100 µm.

Supplementary Movie 5. 3D reconstruction of the F-actin staining on a convex sinusoidal cylinder substrate on day 8. Scale bar is 100 µm.

Supplementary Movie 6. 3D reconstruction of the F-actin and DNA staining on a concave spherical substrate on day 8, for cells treated with TGF-β, with a specific focus on the formation of cell bridges underneath the detached cell sheet. Scale bar is 100 µm

Supplementary Movie 7. 3D reconstruction of the F-actin and DNA staining on a convex spherical substrate on day 8, highlighting the reorientation of stress fibre subpopulations on the saddle-shaped transition between two hemispheres. Scale bar is 100 µm.

Supplementary Movie 8. Animated z-stack of the F-actin and DNA staining on a convex spherical substrate on day 8, highlighting the reorientation of stress fibre subpopulations between the hemispheres. Scale bar is 100 µm.

Supplementary Movie 9. 3D reconstruction of the F-actin and DNA staining on a concave spherical substrate at 200% scale on day 8, showing a central hole in the incomplete cell sheet and the presence of anchoring cell bridges underneath the cell sheet. Scale bar is 100 µm.
