# Supplementary figures and images for: Emergent collective organization of bone cells in complex curvature fields
Source: Nat Commun. 2023 Mar 3;14:855. doi: 10.1038/s41467-023-36436-w (PMC9984480; doi:10.1038/s41467-023-36436-w)

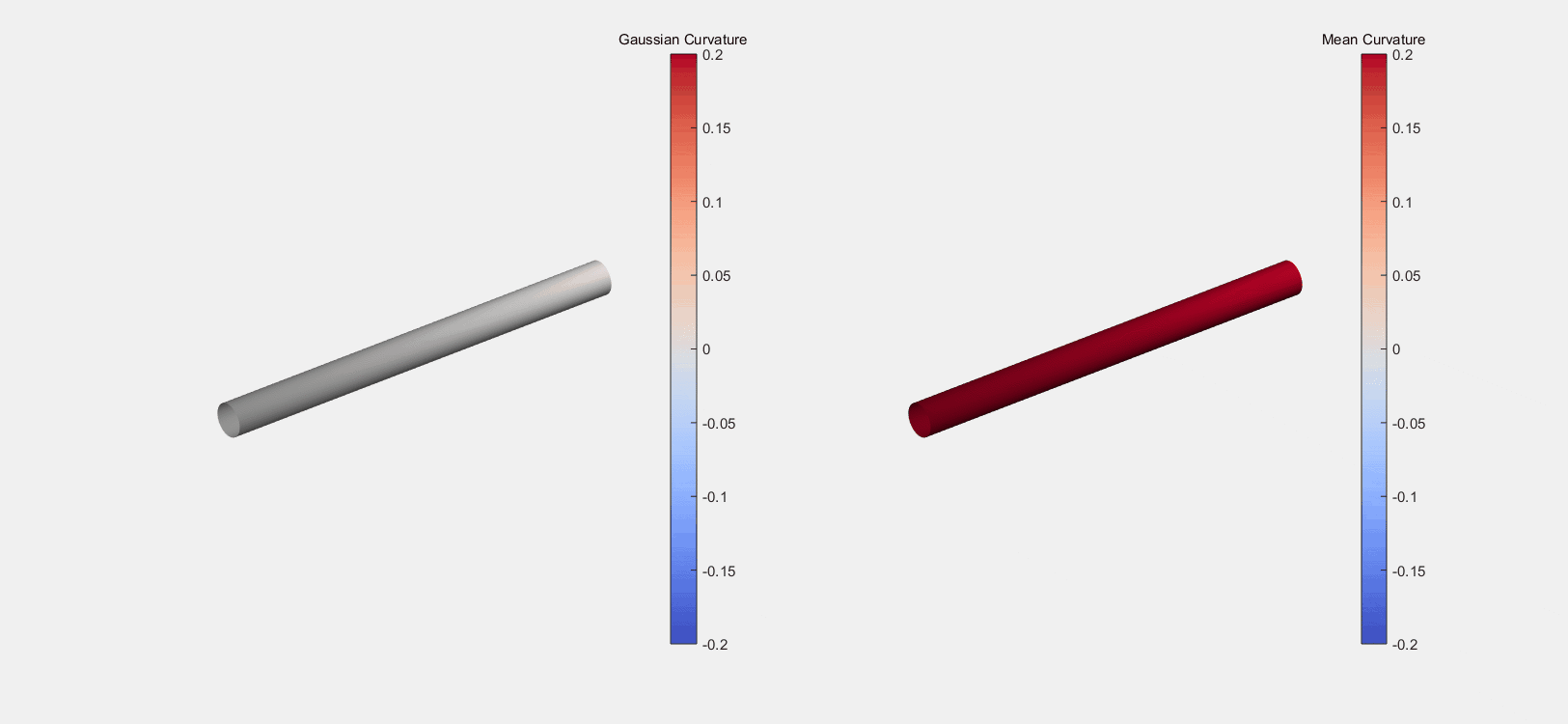

Supplement: Supplementary file 3 — Supplementary Video 1 [file 41467_2023_36436_MOESM3_ESM.gif]

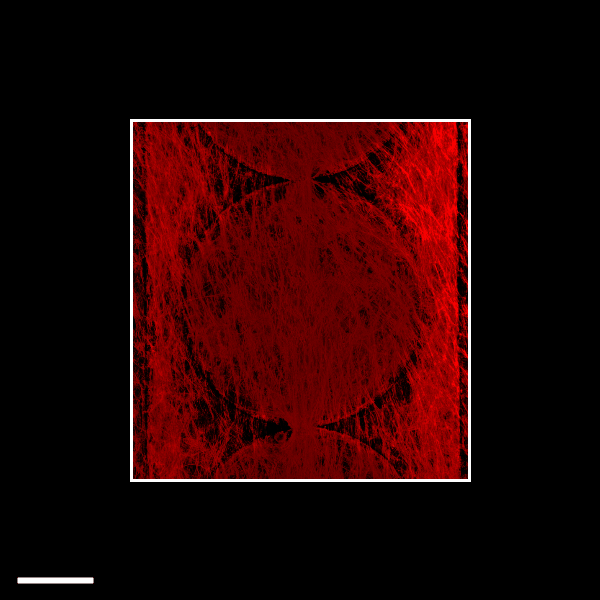

Supplement: Supplementary file 4 — Supplementary Video 2 [file 41467_2023_36436_MOESM4_ESM.gif]

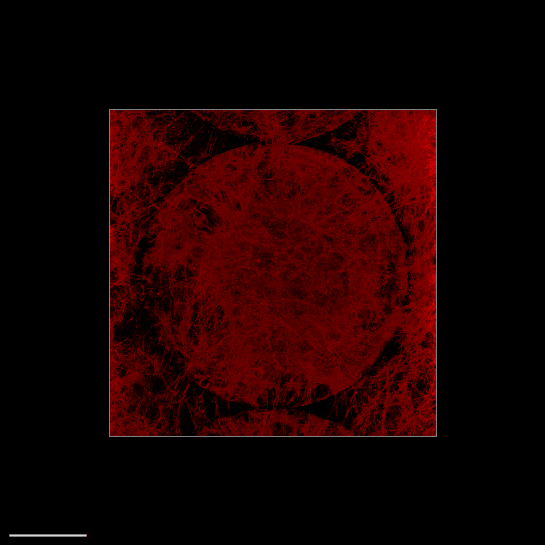

Supplement: Supplementary file 5 — Supplementary Video 3 [file 41467_2023_36436_MOESM5_ESM.gif]

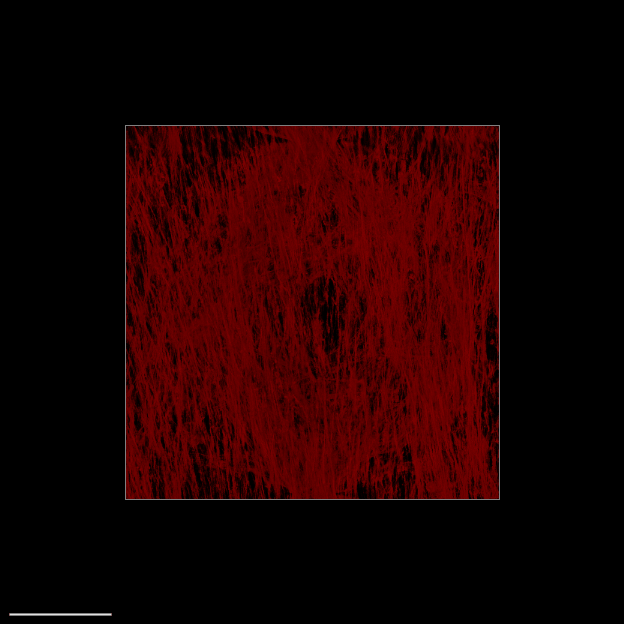

Supplement: Supplementary file 6 — Supplementary Video 4 [file 41467_2023_36436_MOESM6_ESM.gif]

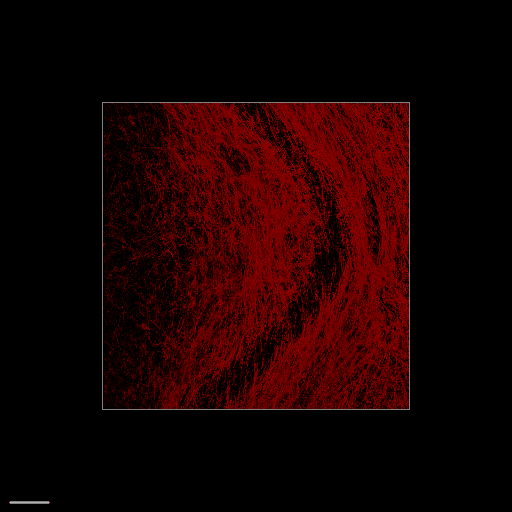

Supplement: Supplementary file 7 — Supplementary Video 5 [file 41467_2023_36436_MOESM7_ESM.gif]

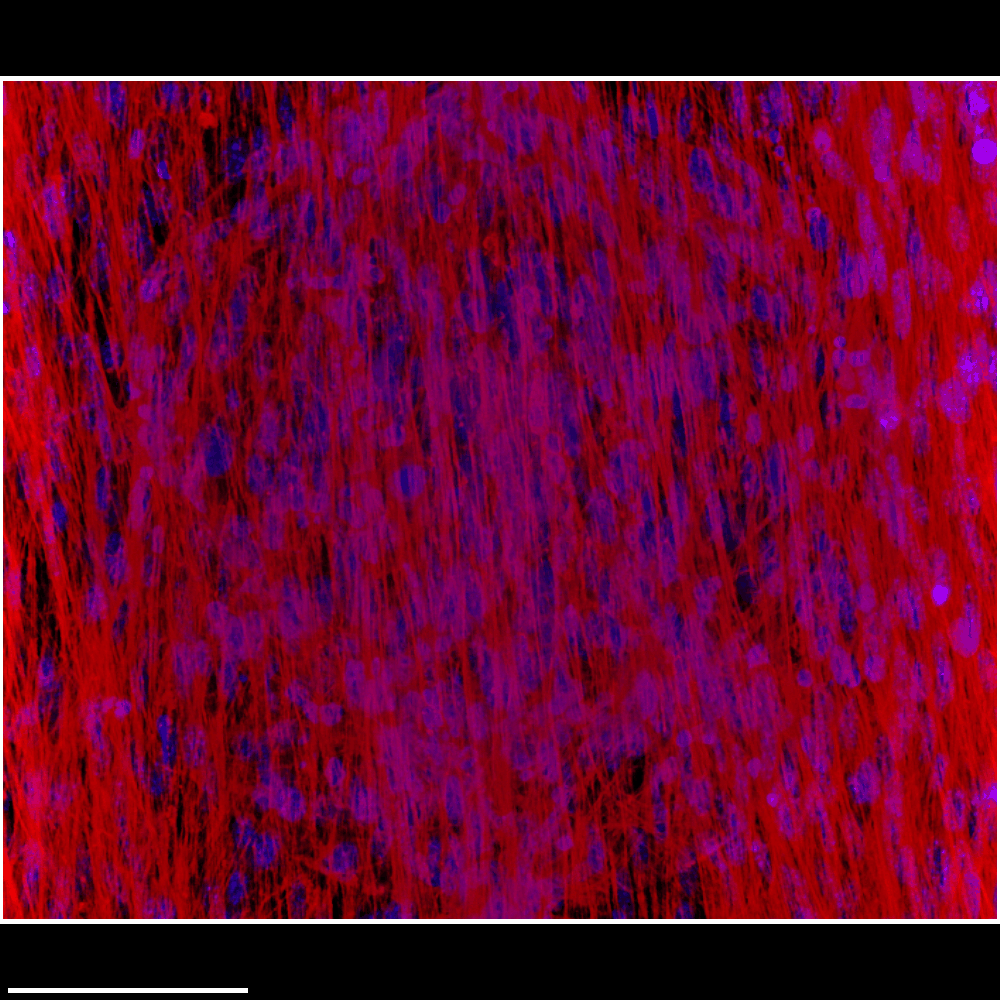

Supplement: Supplementary file 8 — Supplementary Video 6 [file 41467_2023_36436_MOESM8_ESM.gif]

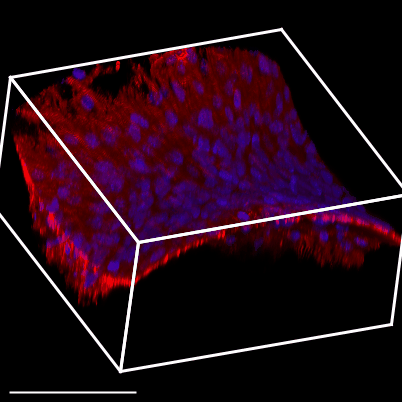

Supplement: Supplementary file 9 — Supplementary Video 7 [file 41467_2023_36436_MOESM9_ESM.gif]

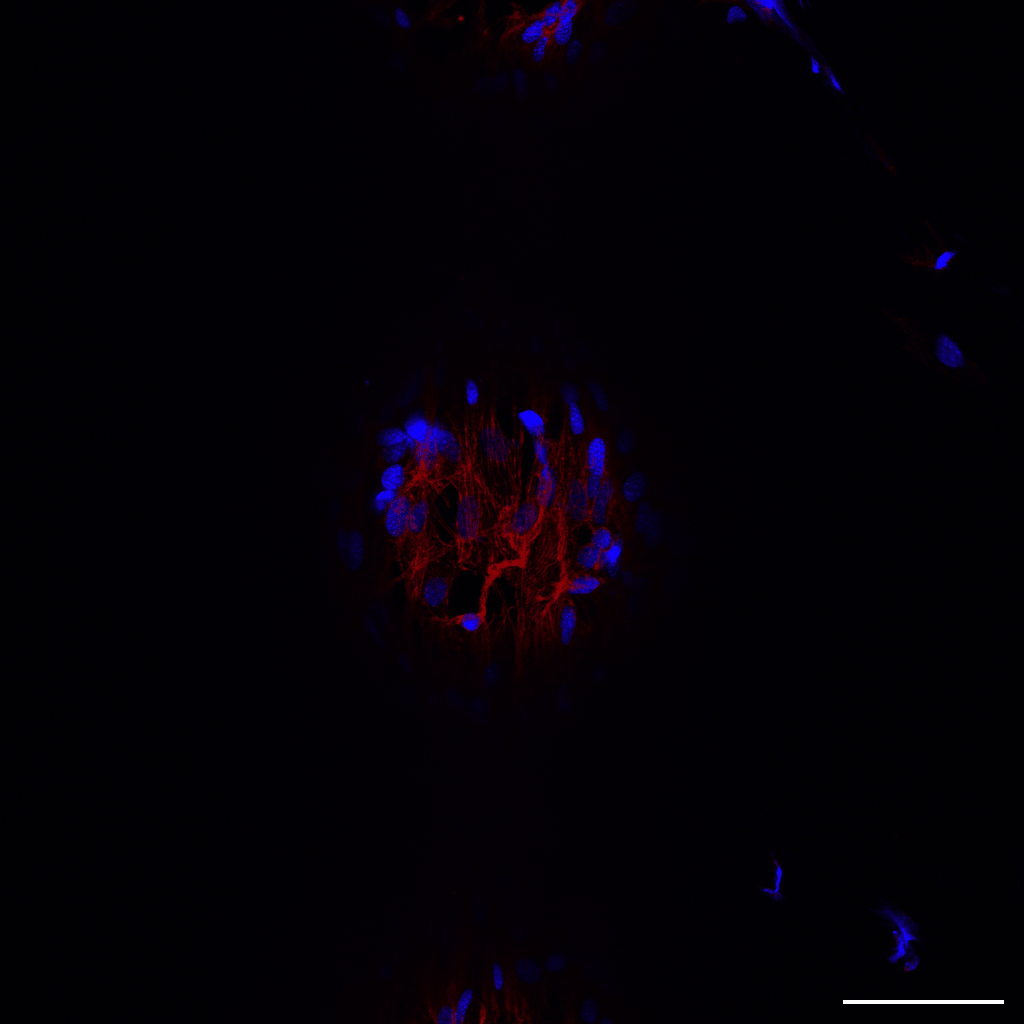

Supplement: Supplementary file 10 — Supplementary Video 8 [file 41467_2023_36436_MOESM10_ESM.gif]
