# Supplementary material for: Emergent collective organization of bone cells in complex curvature fields
Source: Nat Commun. 2023 Mar 3;14:855. doi: 10.1038/s41467-023-36436-w (PMC9984480; doi:10.1038/s41467-023-36436-w)
Supplement: Supplementary file 12 — Reporting Summary [file 41467_2023_36436_MOESM12_ESM.pdf]

nature portfolio

Corresponding author(s): S. Callens

Last updated by author(s): Jan 18, 2023

Reporting Summary

Nature Portfolio wishes to improve the reproducibility of the work that we publish. This form provides structure for consistency and transparency in reporting. For further information on Nature Portfolio policies, see our Editorial Policies and the Editorial Policy Checklist.

Statistics

For all statistical analyses, confirm that the following items are present in the figure legend, table legend, main text, or Methods section.

n/a	Confirmed	
☐	☒	The exact sample size (*n*) for each experimental group/condition, given as a discrete number and unit of measurement
☐	☒	A statement on whether measurements were taken from distinct samples or whether the same sample was measured repeatedly
☐	☒	The statistical test(s) used AND whether they are one- or two-sided<br>*Only common tests should be described solely by name; describe more complex techniques in the Methods section.*
☒	☐	A description of all covariates tested
☐	☒	A description of any assumptions or corrections, such as tests of normality and adjustment for multiple comparisons
☐	☒	A full description of the statistical parameters including central tendency (e.g. means) or other basic estimates (e.g. regression coefficient) AND variation (e.g. standard deviation) or associated estimates of uncertainty (e.g. confidence intervals)
☐	☒	For null hypothesis testing, the test statistic (e.g. *F*, *t*, *r*) with confidence intervals, effect sizes, degrees of freedom and *P* value noted<br>*Give P values as exact values whenever suitable.*
☒	☐	For Bayesian analysis, information on the choice of priors and Markov chain Monte Carlo settings
☒	☐	For hierarchical and complex designs, identification of the appropriate level for tests and full reporting of outcomes
☐	☒	Estimates of effect sizes (e.g. Cohen's *d*, Pearson's *r*), indicating how they were calculated

Our web collection on statistics for biologists contains articles on many of the points above.

Software and code

Policy information about availability of computer code

Data collection	Most confocal images were acquired using NIS-Elements software (version 4.51.01). A few images were acquired using LAS AF software (version 2.7.3).
Data analysis	Custom code was developed in Matlab (versions 2018b and 2020a) for analysis of the confocal microscopy data. In some cases, the open-source software Fiji (version 1.53c) was used to prepare microscopy images for visualisation in the manuscript (e.g. representative maximum intensity projections) or to prepare the data for further analysis in Matlab. For segmentation of the 3D meshes in Figure 4, the open-source software 3D Slicer (version 4.11) was used, and the meshes were cleaned up using the open-source software Meshlab (version 2021.07) and Meshmixer (version 3.5). The surface curvature was estimated using custom codes in Spyder (version 5) with Python (version 3.8). For statistical analysis and the generation of statistics graphs, the software Prism (version 8.4.2) was used.

For manuscripts utilizing custom algorithms or software that are central to the research but not yet described in published literature, software must be made available to editors and reviewers. We strongly encourage code deposition in a community repository (e.g. GitHub). See the Nature Portfolio guidelines for submitting code & software for further information.

Data

Policy information about availability of data

All manuscripts must include a data availability statement. This statement should provide the following information, where applicable:

- Accession codes, unique identifiers, or web links for publicly available datasets
- A description of any restrictions on data availability
- For clinical datasets or third party data, please ensure that the statement adheres to our policy

Data supporting the findings of this study are contained within the manuscript and its Supplementary Information file. Additional microscopy data is available from the corresponding author upon reasonable request. Source data are provided with this paper.

Human research participants

Policy information about studies involving human research participants and Sex and Gender in Research.

Reporting on sex and gender	This research does not involve human research participants.
Population characteristics	This research does not involve human research participants.
Recruitment	This research does not involve human research participants.
Ethics oversight	This research does not involve human research participants.

Note that full information on the approval of the study protocol must also be provided in the manuscript.

Field-specific reporting

Please select the one below that is the best fit for your research. If you are not sure, read the appropriate sections before making your selection.

[x] Life sciences [ ] Behavioural & social sciences [ ] Ecological, evolutionary & environmental sciences

For a reference copy of the document with all sections, see nature.com/documents/nr-reporting-summary-flat.pdf

Life sciences study design

All studies must disclose on these points even when the disclosure is negative.

Sample size	No statistical methods were used to determine sample size prior to the experiments. Sample sizes were based on previous experience in our group with cell-material interactions and other groups with similar experiments. The main limitation on sample size was throughput of fabrication and imaging. At least three biological replicates were performed for all conditions, unless otherwise indicated. The substrates on which cells were grown consisted of repetitive units (periodic substrates) that all showed similar results and for which the data could be aggregated. For specific analyses (such as nuclear centroid position or stress fiber orientation), the information of many thousands of cells was correlated to the local surface curvature they were experiencing, resulting in sufficiently high numbers for statistical analysis. All the information on sample sizes and statistics is provided in the figure captions.
Data exclusions	No data were excluded. In case of poor fluorescent staining or damage to the sample prior to imaging, confocal microscopy data was not acquired and the data was not included as an independent experiment.
Replication	The experiments have been repeated at least three times with similar results, as verified by fluorescence widefield or confocal microscopy.
Randomization	The PDMS substrates were randomly assigned to experimental groups (e.g. D5, D8, control medium, supplemented medium,...) and samples were randomly ordered for imaging and analysis. Samples from different groups were imaged using the same conditions and analysed using the same codes.
Blinding	Investigators were not blinded during cell culture experiments as they needed to know the appropriate treatment to provide to the cells. The investigators could not be blinded from the type of substrate geometry under investigation, as it became immediately obvious during confocal microscopy. Samples were labelled with numbers during microscopy (thus being blinded from which condition was imaged). Confocal microscopy was partially blinded, with image parameters being defined on a small region of the substrate after which the tiling function was used to image the rest of the substrate automatically. For data analysis using image processing codes, blinding was not relevant as all quantification was performed automaticaly and objectively using the same codes.

Reporting for specific materials, systems and methods

We require information from authors about some types of materials, experimental systems and methods used in many studies. Here, indicate whether each material, system or method listed is relevant to your study. If you are not sure if a list item applies to your research, read the appropriate section before selecting a response.

Materials & experimental systems

n/a	Involved in the study
☐	☒ Antibodies
☐	☒ Eukaryotic cell lines
☒	☐ Palaeontology and archaeology
☒	☐ Animals and other organisms
☒	☐ Clinical data
☒	☐ Dual use research of concern

Methods

n/a	Involved in the study
☒	☐ ChIP-seq
☒	☐ Flow cytometry
☒	☐ MRI-based neuroimaging

Antibodies

Antibodies used	anti-Col1 (1:500, recombinant rabbit polyclonal, Sigma-Aldrich sab4500362)<br>anti-RUNX2 (1:500 recombinant rabbit monoclonal (EPR14334), Abcam ab192256)<br>anti-FN (1:250, recombinant Alexa Fluor 488 conjugated rabbit monocolonal (EPR19241-46), Abcam ab237286)<br>Donkey anti-Rabbit IgG secondary ab (1:500, Alexa Fluor 488 conjugated polyclonal, Thermo-Fisher A-21206)
Validation	Antibodies were validated by manufacturers for IHC/IF in mouse cells, and are commonly used in the literature.<br>anti-Col1 (sab4500362): https://www.sigmaaldrich.com/GB/en/product/sigma/sab4500362 , e.g. used in https://doi.org/10.1186/s13287-022-03072-y<br>anti-RUNX2 (ab192256): https://www.abcam.com/runx2-antibody-epr14334-ab192256.html, e.g. used in: https://doi.org/10.1038/s41598-021-85415-y<br>anti-FN (ab237286): https://www.abcam.com/alexa-fluor-488-fibronectin-antibody-epr19241-46-ab237286.html, tested on fixed Hepa1-6 (mouse hepatoma epithelial cells).<br>Donkey anti-Rabbit secondary ab (A-21206): https://www.thermofisher.com/antibody/product/Donkey-anti-Rabbit-IgG-H-L-Highly-Cross-Adsorbed-Secondary-Antibody-Polyclonal/A-21206, e.g. used in: doi: 10.7554/eLife.67624.

Eukaryotic cell lines

Policy information about cell lines and Sex and Gender in Research

Cell line source(s)	Commercially available murine cell line (MC3T3-E1) obtained from Sigma-Aldrich (ECACC). For a few experiments (reported in Supplementary Figures 7-9), MC3T3-E1 cells obtained from ATCC were used.
Authentication	Cells were authenticated originally by ECACC or ATCC, and not further authenticated as part of this study.
Mycoplasma contamination	The cells were tested for mycoplasma contamination and tested negative.
Commonly misidentified lines (See ICLAC register)	No commonly misidentified cell lines were used.
